# Impact of diet on inflammatory bowel disease risk: systematic review, meta-analyses and implications for prevention

**DOI:** 10.1016/j.eclinm.2025.103353

**Published:** 2025-07-14

**Authors:** Antoine Meyer, Manasi Agrawal, Einat Savin-Shalom, Emily C.L. Wong, Carrie Levinson, Stephanie Gold, Neeraj Narula, Jean-Frédéric Colombel, Franck Carbonnel

**Affiliations:** a Service de Gastroentérologie, University Hospital of Bicêtre, Assistance Publique-Hôpitaux de Paris, Le Kremlin Bicêtre, France; b Faculté de Médecine, Université Paris Saclay, Le Kremlin Bicêtre, France; c INSERM 1018, INSERM, UPS, UVSQ Institut Gustave Roussy, 114 rue Edouard Vaillant, 94805, Villejuif Cedex, France; d The Dr. Henry D. Janowitz Division of Gastroenterology, Icahn School of Medicine at Mount Sinai, New York, NY, USA; e Department of Environmental Medicine and Climate Science, Icahn School of Medicine at Mount Sinai, New York, NY, USA; f Center for Molecular Prediction of Inflammatory Bowel Disease, Department of Clinical Medicine, Aalborg University, Copenhagen, Denmark; g Department of Medicine (Division of Gastroenterology) and Farncombe Family Digestive Health Research Institute, McMaster University, Hamilton, ON, Canada; h Levy Library, Icahn School of Medicine at Mount Sinai, New York, NY, USA

**Keywords:** Inflammatory bowel disease, Crohn's disease, Diet, Ultra-processed foods, Prevention

## Abstract

**Background:**

Data on dietary risk factors for inflammatory bowel disease (IBD), while extensive, are inconsistent. Our aim was to systematically review and meta-analyze available data unraveling the relationship between diet and IBD subtypes, Crohn's disease (CD) and ulcerative colitis (UC).

**Methods:**

We conducted a systematic literature review following PRISMA guidelines, from inception to May 8 2025, using OVID Medline, Embase, and Scopus databases, to identify prospective cohorts of healthy participants, on the association between diet and the risk of CD or UC. Meta-analyses were performed using random-effects model, pooling hazard ratios (HRs) for each exposure category, relative to the lowest.

**Findings:**

Of 7916 studies identified by the search, 72 studies (65 in adults, 7 in children) met the inclusion criteria. The 65 adult cohort studies included 2.043.601 participants; 62.3% were women, the mean age at recruitment was 53.1 years and mean follow up was 12.8 years. Overall, 1902 participants developed CD and 4617 developed UC. Inflammatory diet (pooled aHR 1.63, 95% CI: 1.26, 2.11) and ultra-processed foods (pooled aHR 1.71, 95% CI: 1.36–2.14) were associated with an increased risk of CD. High fiber intake (pooled aHR 0.53, 95% CI: 0.41–0.70), Mediterranean diet (pooled aHR 0.59, 95% CI: 0.43–0.81), healthy diet (pooled aHR 0.70, 95% CI: 0.54–0.91), and unprocessed or minimally processed foods (pooled aHR 0.71, 95% CI: 0.53–0.94) were associated with a lower risk of CD. No consistent associations were found between individual foods or food patterns and the risk of UC.

**Interpretation:**

This study summarizes evidence on the link between specific dietary items or patterns and the risk of IBD. These data will help inform the design of prevention trials that include a dietary component as well as prevention strategies overall.

**Funding:**

This study received no funding

**Research in context**

**Evidence before this study**

The role of environmental factors in IBD risk, particularly diet, is being increasingly recognized; yet, the data produced by observational studies are heterogenous and often inconsistent. We conducted a systematic literature review following PRISMA guidelines, from inception to May 8th, 2025, using OVID Medline, Embase, and Scopus databases. We included studies that met the following criteria: (1) prospective cohort studies or case–control studies nested in prospective cohorts, (2) prospective assessment of diet in children or adults prior to CD or UC diagnosis, (3) reporting of the outcome of IBD, CD or UC diagnosis during follow-up, and (4) study of an association between food exposure and the risks of IBD, CD or UC. We excluded review articles, meta-analyses and, given the risk of recall bias, retrospective case–control studies. Meta-analyses were performed using random-effects model, pooling hazard ratios (HRs) for each exposure category, relative to the lowest.

**Added value of this study**

In this systematic literature review and in meta-analyses based on 72 prospective observational studies, we found that inflammatory and ultra-processed diets were associated with a higher risk of CD, while Mediterranean, healthy diets, unprocessed/minimally processed foods, and high fiber intakes were associated with a lower risk of CD. No consistent associations were found between individual foods or food patterns and the risk of UC.

**Implications of all the available evidence**

This paper adds further evidence and consistency to the role of diet in the risk of CD. As diet is a modifiable lifestyle factor, these data are not only relevant for the understanding of IBD but also for building future prevention recommendations and trials.

## Introduction

The incidence and prevalence of inflammatory bowel diseases (IBD), including subtypes Crohn's disease (CD) and ulcerative colitis (UC) are increasing globally.^1^^,^^2^ Currently, there is no cure for IBD. Crohn's disease and UC cause substantial morbidity and complications.^3^^,^^4^ Patients affected with these diseases need continuous follow-up and often, costly maintenance treatments.^5^

While the etiology of IBD is not yet understood, increases in IBD incidence and prevalence have paralleled environmental shifts. The role of environmental factors in IBD risk, particularly diet, is being increasingly recognized.^6^ As diet is a modifiable lifestyle factor, these data are not only relevant for the understanding of IBD but also for building future prevention studies. Yet, the data produced by observational studies are heterogenous and often inconsistent. Therefore, in this study, we systematically reviewed and meta-analyzed available data on pre-illness diet and IBD risk.

## Methods

### Search strategy and selection criteria

This study was conducted according to the Preferred Items for Systematic Reviews and Meta-Analyses (PRISMA) statement,^7^ using the Covidence platform.

We conducted the comprehensive search in three electronic databases, OVID Medline, Embase and Scopus from inception to May 8, 2025, to identify studies on the association between dietary variables and the risk of IBD, CD or UC (Supplementary Table S1). We did not apply any language restriction.

For the systematic literature review, we included studies that met the following criteria: (1) prospective cohort studies or case–control studies nested in prospective cohorts, (2) prospective assessment of diet in children or adults prior to CD or UC diagnosis, (3) reporting of the outcome of IBD, CD or UC diagnosis during follow-up, and (4) study of an association between food exposure and the risks of IBD, CD or UC. We excluded review articles, meta-analyses and, given the risk of recall bias, retrospective case–control studies.

### Data extraction and risk of bias assessment

All abstracts were independently screened by two authors (FC, ESS) with review by a third arbitrator in case of discrepancy or disagreement (AM). The data and risk of bias was assessed independently by three reviewers (FC, ESS, AM) using the STROBE criteria.^8^ Disagreements were resolved by discussion and involved a fourth author, when necessary.

### Meta analyses

We performed meta-analyses of studies that reported associations between individual foods or food patterns, and CD or UC in at least two studies. As there are differences in CD and UC risk factors and pathogenesis, we meta-analyzed data for CD or UC reported separately, but not IBD overall. Using the generic inverse-variance method with random-effect meta-analysis, we combined individual reported hazard ratios (HR) and 95% confidence interval (95% CI) into a pooled-HR (95% CI) for the highest category, relative to the lowest category of exposure from the fully adjusted models (or if unavailable, from the crude models). If HR were not available, risk ratio (RR) or odds ratio (OR) were used. The HR is an instantaneous RR and as the outcomes CD and UC are rare events, the OR would approximate the RR.^9^ We used the random-effects model as this provides a more conservative estimate than a fixed effects model, when there is heterogeneity. Within each meta-analysis, when there were several studies from the same cohort, we included the study with the largest sample size or the most recent study. In the meta-analysis, the results of one study^10^ were not included, as the hazard ratios presented for a one-point intake increase were not convertible into hazard ratios for high vs. low consumption (e.g. quartiles) with the available data. We assessed heterogeneity using the chi-squared test and I^2^ values. The chi-square test suggests heterogeneity between studies when the p-value is less than 0.10.^11^ The I^2^ test describes the proportion of variability in effect estimates that is due to heterogeneity rather than chance For I^2^ values below 40%, heterogeneity might not be important; between 30% and 60% heterogeneity may be moderate; between 50% and 90% it is substantial, and above 75% it is considerable.^11^ We used funnel plots and calculated Egger's regression intercept to assess publication bias when there were ten or more studies for each category of exposure and outcome.^12^ Analyses were performed with R statistical software (version 4.2.2) and the “meta” R package.^13^

There was funding source for this study.

## Results

### Identification of studies

**Fig. 1:**
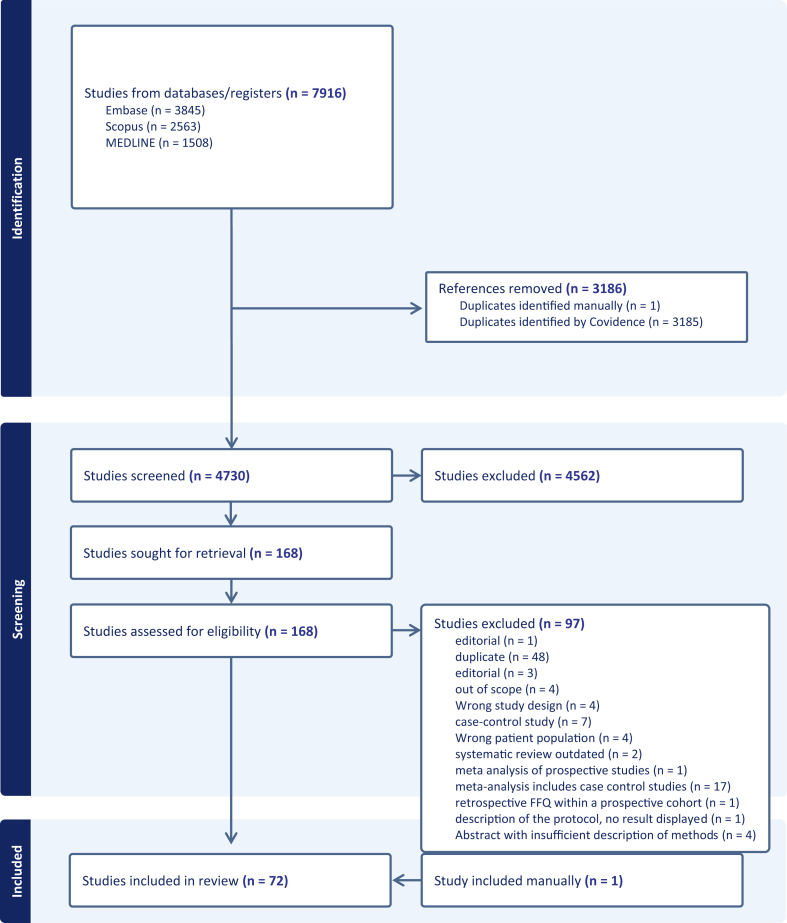
Flow PRISMA chart.

The literature search protocol is available in the Supplementary Data. The database search identified 7916 studies. After removing duplicates, 4730 articles were screened, based on titles and abstracts. Of these, 4562 studies were not relevant to the research question. Of the remaining, we excluded 97 studies. One relevant paper was added manually.^14^ Finally, 72 studies published between 2008 and 2025 were included in the systematic review (Fig. 1).

### Study characteristics

The study characteristics (including cohort sizes, gender distributions and mean follow-up durations etc.) are outlined in Supplementary Table S1. Quality of studies (including risks of bias), was assessed using STROBE criteria; they are shown in a separate, Supplementary File. Unless otherwise specified, quality of the studies was adequate. Of the 72 studies included, 15 were case–control studies nested in prospective cohorts and 57 were cohort studies. Sixty-five studies were conducted in adults and seven in children. Fifty-one studies were conducted in Europe (including 21 from European Prospective Investigation into Cancer (EPIC), 17 from United Kingdom Biobank, 4 from Swedish adult cohorts and 6 from Scandinavian cohort of babies), 17 studies were conducted in the United States (within the Nurse's Health Studies (NHS) I and II and Health Professional follow-up study), two studies were based upon the multinational prospective urban rural epidemiology (PURE) cohort, one study was performed in Israel and another one in China. A total of 2,043,601 participants of the main cohorts were included in the most recent articles or those with the largest sample size (see Supplementary Table S2), of whom 62.3% were women (range 44%–82%). The mean age at recruitment was 53.1 years (range 43–61), with a mean follow-up of 12.8 years (range 2.3–24.5). Of these participants, 1902 developed Crohn's disease (CD) and 4617 developed ulcerative colitis (UC).

In most studies, assessments of dietary intakes were based on country-specific, validated food-frequency questionnaires (FFQ; Supplementary Table S1). The EPIC and Swedish adult studies were based on baseline questionnaires; those conducted within the NHS and UK biobanks relied on baseline and follow-up questionnaires.

**Fig. 2:**
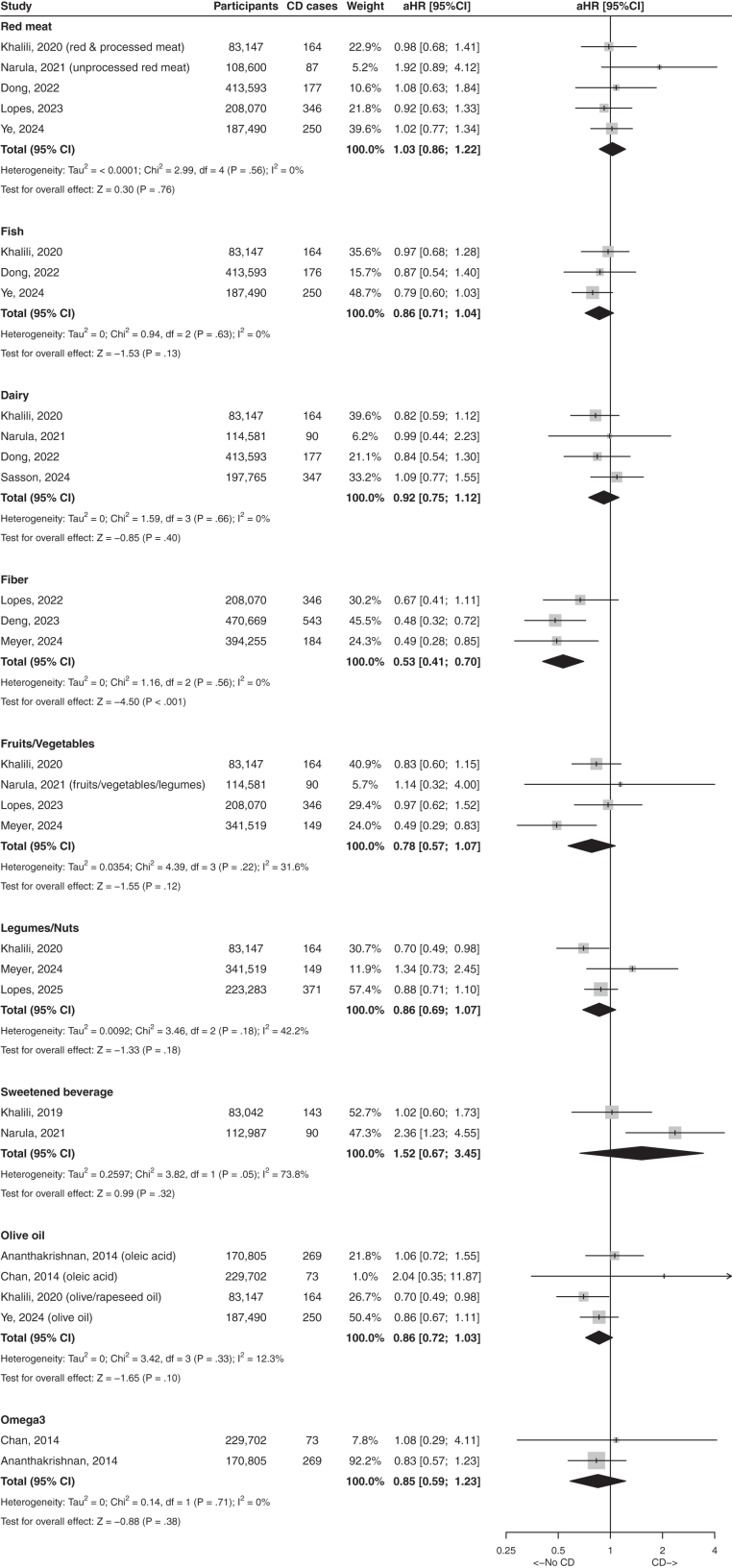
Forest plot with studies reporting association between food items and risks of Crohn's disease. Results present highest quantile compared with lowest quantile. aHR, adjusted hazard ratio; CD, Crohn's disease; CI, confidence interval.

**Fig. 3:**
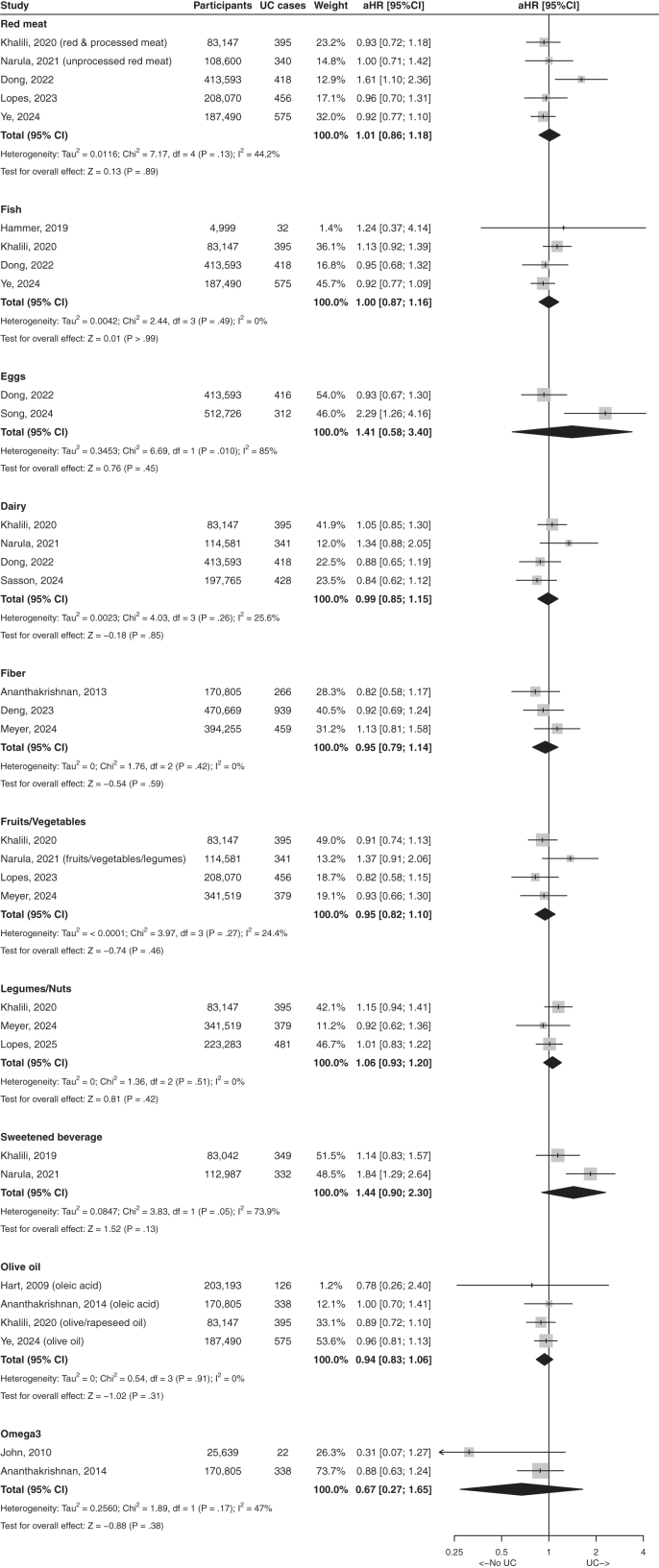
Forest plot with studies reporting association between food items and risks of ulcerative colitis. Results present highest quantile compared with lowest quantile. aHR, adjusted hazard ratio; UC, ulcerative colitis; CI, confidence interval.

**Fig. 4:**
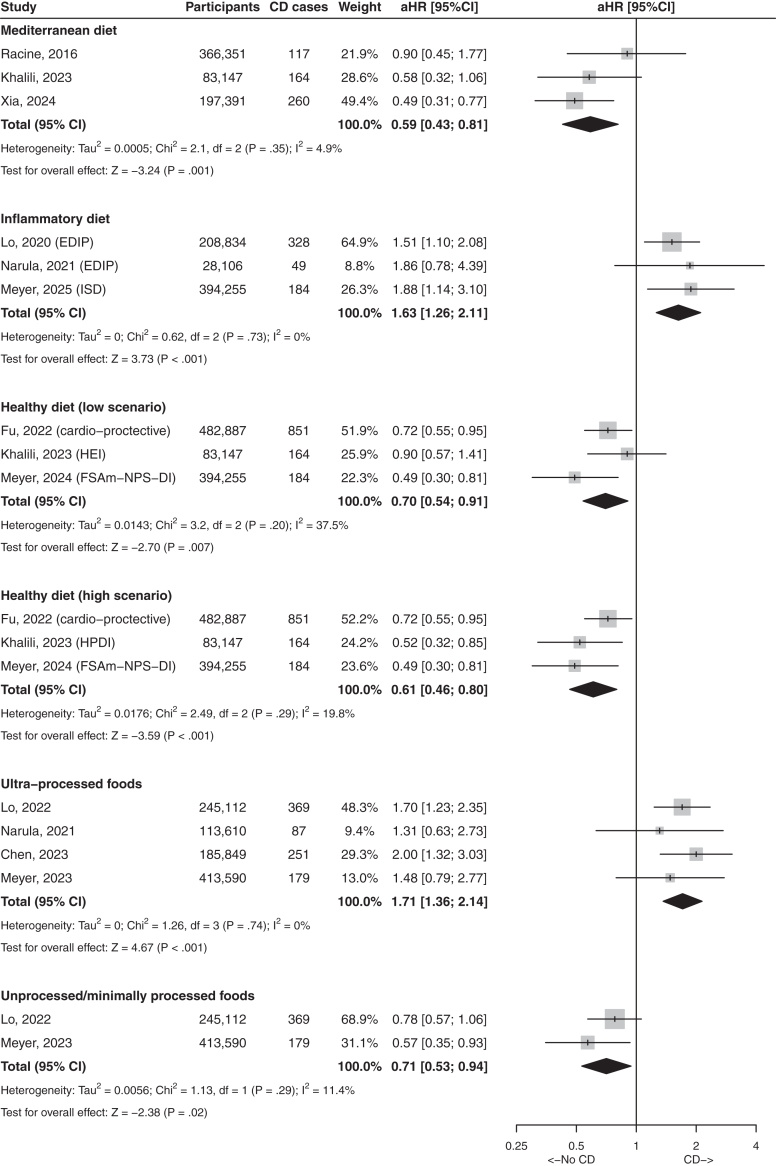
Forest plot with studies reporting association between food patterns and risks of Crohn's disease. Results present highest quantile compared with lowest quantile. aHR, adjusted hazard ratio; CD, Crohn's disease; CI, confidence interval.

**Fig. 5:**
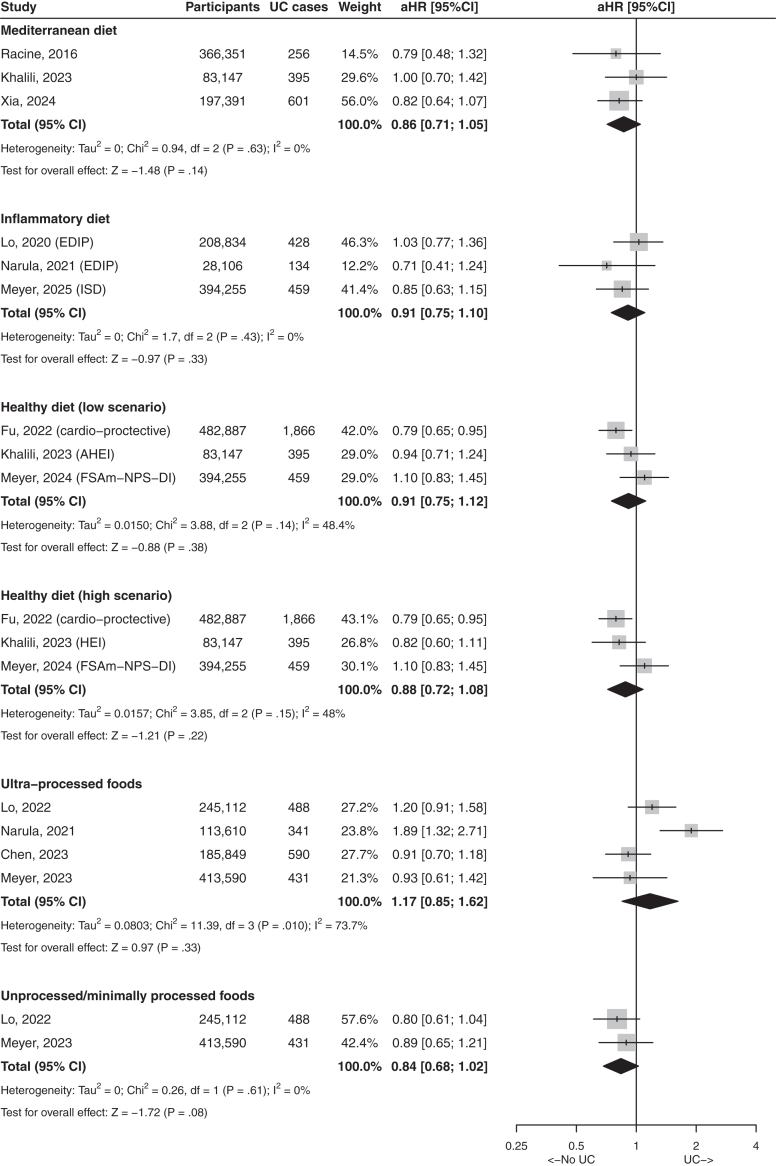
Forest plot with studies reporting association between food patterns and risks of ulcerative colitis. Results present highest quantile compared with lowest quantile. aHR, adjusted hazard ratio; UC, ulcerative colitis; CI, confidence interval.

**Fig. 6:**
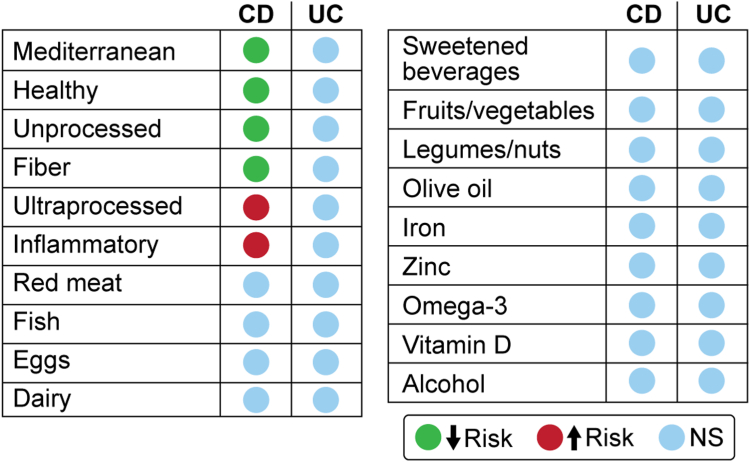
Summary of the meta-analyses of 64 prospective cohort studies which examined the association between pre-disease diet and risk of Crohn's disease (CD) and ulcerative colitis (UC).

Extracted data are included in Supplementary Tables S3–S14, respectively. We present the results in 12 categories, including six foods and nutrients categories, five dietary patterns and one for pregnancy and early life diet. Eighteen meta analyses of 28 articles were performed for CD and 20 meta analyses of 35 articles were performed for UC. All meta analyses (including pooled estimates and I^2^) are shown in Fig. 2, Fig. 3, Fig. 4, Fig. 5, Fig. 6, in Supplementary Figures S1–S4 and, when significant, in the text.

### Foods and nutrients

#### Protein and protein-rich foods

We identified 15 relevant articles on the association of protein and protein-rich foods and the risk of IBD, CD and UC (Supplementary Table S3).^15^, ^16^, ^17^, ^18^, ^19^, ^20^, ^21^, ^22^, ^23^, ^24^, ^25^, ^26^, ^27^, ^28^, ^29^ Separate meta-analyses were performed for each of the following foods: red meat, fish, eggs, and dairy. Null associations were found with the outcomes of CD and UC (Figs. 2 and 3).

#### Carbohydrates

We identified 15 studies that reported on the association of carbohydrates and the risk of IBD (Supplementary Table S4).^16^, ^17^, ^18^, ^19^^,^^25^^,^^26^^,^^29^, ^30^, ^31^, ^32^, ^33^, ^34^, ^35^, ^36^, ^37^ In the meta-analysis, fiber consumption was associated with a lower risk of CD (pooled aHR 0.53, 95% CI: 0.41–0.70, I^2^ = 0%) but not UC (pooled aHR 1.01, 95% CI: 0.81–1.25, I^2^ = 0%). Fruits/vegetables or legumes/nuts intakes were not associated with either CD or UC risk (Figs. 2 and 3). There was no association between sweetened beverages and CD or UC (Figs. 2 and 3); the study by Fu T et al.,^37^ was not included in this meta-analysis because it was the only one to assess separately artificially-sweetened beverages and sugar-sweetened.

#### Dietary fat and fatty acids

The impact of dietary fat and fatty acids on the risk of IBD was reported in 14 studies (Supplementary Table S5).^16^^,^^18^^,^^19^^,^^23^^,^^26^^,^^28^^,^^29^^,^^33^^,^^38^, ^39^, ^40^, ^41^, ^42^, ^43^ On meta-analysis, there was no association between olive oil, n-3 fatty acids and CD or UC. DHA intake was associated with a lower risk of UC and CD in three overlapping EPIC studies (Figs. 2 and 3).^38^^,^^40^^,^^42^ The two studies based on the NHS cohorts assessed the n-3/n-6 ratio^19^^,^^41^; the largest one found no association with UC.^41^ Fish oil was associated with a lower risk of UC and CD^28^^,^^43^ in two overlapping UK biobank studies. Fried foods were associated with a higher risk of UC in the PURE study.^29^

#### Micronutrients

Associations between micronutrients and risk of CD and UC are shown in Supplementary Table S6 and Supplementary Figures S1 and S2. Two studies assessed the association between iron intake and the risk of IBD^15^^,^^33^; the meta-analysis showed no association between iron intake and UC risk (Supplementary Figure S2). The only study on iron intake and CD risk found no significant association.^15^ The meta-analysis of the two studies that evaluated dietary zinc intake in relation with CD or UC risks found null association (Supplementary Figures S1 and S2).^44^^,^^45^ Two studies investigated total sodium intake^29^^,^^46^ and found no association with the risk of IBD. The consumption of salty foods and snacks was associated with an increased risk of IBD,^29^ in the PURE study. One nested case–control study reported that dietary potassium intake was inversely associated with the risk of CD.^46^ Hart et al.^33^ assessed the impact of vitamin C, vitamin E, carotene and retinol, on UC risk and reported null associations. Dietary polyphenols have antioxidant properties; they were studied in an EPIC-based nested case–control study. Total polyphenols were not associated with CD or UC, while flavones and resveratrol were inversely associated with CD (aHR 0.61; 95% CI: 0.28–1.30) and 0.40 (0.20–0.82), respectively).^47^ Another study, based on the UK Biobank, found that total antioxidant capacity was associated with a reduced risk of CD but not UC.^48^ Quercetin and anthocyanin are plant flavonoids from the group of polyphenols. In two studies based on the UK Biobank, higher quercetin intake was associated with a lower risk of UC,^49^ while anthocyanin was associated with a reduced risk of UC, but not CD.^50^ Four studies^33^^,^^51^, ^52^, ^53^ investigated the impact of dietary vitamin D on IBD risk. The meta-analysis showed no association between vitamin D and CD or UC (Supplementary Figures S1 and S2).

#### Alcohol

Two studies based on distinct cohorts investigated alcohol intake (Supplementary Table S7).^54^^,^^55^ In the meta-analysis, overall alcohol consumption was not associated with the risk of CD or UC (Supplementary Figures S1 and S2).^54^^,^^55^ One of these two studies reported that beer was associated with decreased risk of CD, whereas liquor consumption was associated with an increased risk of UC.^55^ Another study reported that red wine consumption was associated with a lower risk of IBD; no such association was observed with other alcoholic beverages.^56^

#### Miscellaneous

One study based on the UK-biobank found no significant association between consumption of advanced glycation end products and IBD.^57^ A study based on the NHS cohorts found no association between gluten intake and the risks of CD or UC.^58^

Three abstracts based on the NHS cohorts were included.^59^, ^60^, ^61^ One study assessed dietary tryptophan intake and found no significant association with IBD risk.^61^ Sulfur microbial diet is high in low-calorie beverages, red and processed meats and low in whole grains and vegetables. Two studies reported an increased risk of CD (not UC), with higher sulfur microbial diet scores.^59^^,^^60^

### Dietary patterns

#### Mediterranean diet

The Mediterranean diet is characterized by high consumptions of vegetables, legumes, fruits, nuts, cereals, fish, olive oil and low consumptions of meat and dairy products. Six studies assessed the impact of Mediterranean diet on the risks of CD and UC (Supplementary Table S9).^10^^,^^23^^,^^26^^,^^62^, ^63^, ^64^ In the meta-analysis, Mediterranean diet was associated with a decreased risk of CD (pooled-aHR 0.59, 95% CI: 0.43–0.81, I^2^ = 4.9%) but not UC (pooled-aHR 0.86, 95% CI: 0.71–1.05, I^2^ = 0%) (Figs. 4 and 5). The definition of Mediterranean diet differed slightly between these three papers and is summarized in Supplementary Table S9 bis.

#### Inflammatory potential of the diet

Food groups have been correlated with circulating markers of inflammation.^65^ Four studies assessed the inflammatory potential of diet in relation with the risk of CD and UC (Supplementary Table S10).^14^^,^^66^, ^67^, ^68^ Two studies used the empirical dietary inflammatory pattern (EDIP) score^66^^,^^67^ and two used the inflammatory score of the diet (ISD).^14^^,^^68^ The meta-analysis showed that inflammatory diet was associated with an increased risk of CD (pooled-aHR 1.63, 95% CI: 1.26–2.11, I^2^ = 0%), but not UC (pooled-aHR 0.91, 95% CI: 0.75–1.10, I^2^ = 0%) (Figs. 4 and 5).

#### Healthy dietary indices

Healthy dietary indices assess how a pattern of food consumption aligns with recommendations for prevention of non-communicable diseases. These indices divide foods in healthy (fruits, vegetables, legumes, whole grains, nuts, fish, etc.) and non-healthy foods (added sugar and salt, trans fat, red and processed meat, etc.). Ten studies assessed healthy dietary indices and the risk of CD and UC (Supplementary Table S11).^10^^,^^18^^,^^25^^,^^63^^,^^64^^,^^69^, ^70^, ^71^, ^72^, ^73^ In the Peters et al. study,^10^ the diagnoses of IBD were based on self-declaration of patients. In addition, the results of this study are presented with exposure modeled as a continuous variable and not as categories. Six papers were based on the UK biobank cohort and were published 3 years apart.^25^^,^^64^^,^^69^, ^70^, ^71^^,^^73^ We performed meta-analyses on three studies, based on the EPIC cohort, the Swedish cohorts and the UK biobank cohort.^18^^,^^25^^,^^63^ To take into-account the differential association between healthy eating index (HEI) and healthful plant-based diet (HPDI) with CD,^63^ we performed a meta-analysis with the HEI and another one with the HPDI. In both scenarios, healthy dietary indexes were associated with a decreased risk of CD (pooled-aHR 0.70, 95% CI: 0.54–0.91, I^2^ = 37% and pooled-aHR 0.61, 95% CI: 0.46–0.80, I^2^ = 20%, respectively); no association was observed with UC (Figs. 4 and 5).

#### Processed and ultra processed foods

Five studies have assessed food processing as a risk factor of IBD (Supplementary Table S12).^29^^,74–77^ The reporting quality was adequate although in one study, the follow-up was limited to 2.3 ± 2.2 years.^77^ The meta-analysis was based on four studies and showed that UPF were associated with an increased risk of CD (pooled-aHR 1.71, 95% CI: 1.36–2.14, I^2^ = 0%), whereas non or minimally-processed foods were associated with a decreased risk of CD (pooled-aHR 0.71, 95% CI: 0.53–0.94, I^2^ = 11%); no association was observed with UC (Figs. 4 and 5).

#### A posteriori dietary patterns

In these studies, dietary patterns were generated by principal component analysis, with no *a priori* hypothesis. Five papers matched the criteria of selection (Supplementary Table S13).^10^^,^^21^^,^^45^^,^^62^^,78^ One study, based on the NHS cohorts, was an abstract communicated in 2014.^78^ In the study by Racine et al.,^62^ a pattern characterized by high consumptions of sugar and soft drinks, and low consumptions of vegetables and non-processed seafood was associated with the risk of UC. In the study by Peters et al.,^10^ a “carnivorous pattern” was associated with an increased risk of UC whereas a western dietary pattern was associated with CD risk. The study based on the NHS cohorts found no association between Western or prudent diet and the IBD risk.^78^ The study by Vasseur et al.^45^ found three patterns: “healthy”, “traditional”, “western,” none of which was associated with IBD risk. The study performed by Song et al.,^21^ in China, found that a pattern with high intake of wheat and a low intake of rice and another one including animal-origin foods and fruit intakes were associated with UC. The meta-analysis was not feasible for *a posteriori* dietary patterns.

### Diet during pregnancy and early childhood

Seven studies assessed the effect of diet during pregnancy or early childhood on the risk of developing IBD in the offspring or later in life, respectively (Supplementary Table S14).^79–85^ Two studies found no association between breastfeeding duration and CD or UC.^81,82^ Three studies based on the same Swedish and Norwegian cohorts were published in 2024.^79,80,83^ Approximately 80,000 babies were followed from birth (1999–2009) during a median of 16 years, with information on maternal childhood and diet in pregnancy, based on FFQ. A study reported that a high-quality diet at one year was associated with a reduced risk of IBD. In addition, high intakes of fish at 1 and 3 years were associated with a reduced risk of UC, not CD. A low content of sugar-sweetened beverages was associated with a decreased risk of IBD.^83^ The consumption of meat, dairy, fruits, vegetables and sugar-sweetened beverages showed no significant association.^83^ The same group investigated diet diversity at 1 and 3 year and found no association with subsequent IBD.^80^ In pregnant women^79^ diet diversity (but not quality) was associated with a decreased risk of UC in the offspring. Two abstracts from the Danish National Birth Cohort have found that high intakes in lean fish and N-3PUFAs during pregnancy were associated with a decreased risk of IBD in the offspring^85^ while diet diversity during pregnancy was associated with a decreased risk of CD but not UC, in the offspring.^84^

### Sensitivity analyses

For exposures with significant association in the main analysis, we performed sensitivity analyses where exposure had the same categorization (e.g. only in quartiles for ultra-processed foods) which provided consistent results (Supplementary Figures S3 and S4). In addition, among exposures with significant association in the main analysis, all had significant dose–response associations for CD when combining p-trend: p = 0.003 for fibers, p = 0.03 for mediterranean diet, p = 0.001 for inflammatory potential of the diet, p < 0.01 for healthy dietary indices for both scenario, p < 0.001 for ultra-processed foods, and p = 0.004 for non or minimally-processed foods.^86^

## Discussion

In this systematic literature review and in meta-analyses based on 72 prospective studies, we examine the association between pre-disease diet and the risk of IBD. Most associations were observed for dietary patterns, which take into-account synergistic and/or antagonistic interactions between nutrients, and therefore provide a holistic approach to nutrition. They seem better adapted to nutritional epidemiologic investigations of non-communicable diseases.^87^ The main findings were that inflammatory and ultra-processed diets were associated with a higher risk of CD, while Mediterranean, healthy diets, unprocessed/minimally processed foods, and high fiber intakes were associated with a lower risk of CD. No consistent associations were found for other individual food items such as red meat, fish, dairy, fruits/vegetables, or olive oil (Fig. 2) and CD. No consistent associations were found between individual foods or food patterns and the risk of UC.

Most previous meta-analyses on individual food items or patterns are limited by inclusion of retrospective data and use of dietary data following IBD diagnosis, making them prone to recall and reverse causation biases.^88–104^ The meta-analysis on food processing by Narula et al.^105^ was based on prospective studies and is reproduced in the present study.

It can be argued that associations between foods and risks of CD and UC are due to reverse causality. Diagnosis delays can reach up to 10 years in CD, and 5 years in UC. Yet, in high-income countries, median time to diagnosis is of 6.2 (IQR: 5.0–12.3) months for CD and 3.2 months (IQR: 2.2–5.3) for UC.^106^ Only 11% of patients wait more than two years for the diagnosis of CD and UC.^107^ Most studies included in this review addressed potential reverse causality bias by sensitivity analyses, in which the time elapse between dietary questionnaire and IBD diagnosis was extended for two years or more; results were grossly unchanged. In addition, studies conducted in children and pregnant women are not subject to reverse causation. Furthermore, dietary patterns associated with CD are also associated with obesity. As obesity is associated with CD,^108^ the association between dietary patterns and CD could have been mediated by obesity. However, all studies included in the meta analyses of dietary patterns adjusted for BMI and most (9/11) adjusted for energy intakes.

This report summarizes available evidence on the link between diet and the risk of CD. It identifies four dietary patterns associated with a decreased risk of CD and gives an estimate of the effect size. The various diets associated with a reduced risk of CD have similarities but also differences.^109,110^ They could be proposed, based on persons’ preferences and continued or switched to another, according to their short-term effect on biomarkers (microbiota, metabolomics, etc.). The effect sizes of low quality, inflammatory and ultra-processed diets are of the same magnitude as that of smoking for CD.^111^ In the general population, where a risk of CD approximates 0.5%, having a diet with a protective hazard ratio of CD of 0.6, the number needed to prevent a single CD case would be 500 persons.^112^ In first-degree relatives of patients with CD within simplex and multiplex families, where the CD risk approximates 2% and 10% after 10 years of follow-up^113^, the number needed to prevent a CD case with this diet would be 125 and 25 persons, respectively. Currently, dietary trials are ongoing in first-degree relatives of patients (NCT05211518, NCT05566587, NCT03950336) using microbiota, intestinal permeability or fecal calprotectin as endpoints. Aside from IBD, Mediterranean or healthy diets might decrease the risk of other non-communicable diseases, and should therefore be considered in the general population. Noticeably, the effect sizes of diets associated with CD are of the same magnitude as those of cardiovascular diseases. For Mediterranean diet, pooled HRs are 0.68 [0.51; 0.89] and 0.70 [0.62; 0.80], for CD and coronary heart disease incidences, respectively.^114^ The information collected here could inform diet-based trials and dietary recommendations aimed at preventing CD.

Theoretically, Mendelian randomization are less prone to confounding than observational epidemiology studies. They have shown an inverse association with genetically-predicted serum levels of vitamin D and lycopene, while there are conflicting data about N-3 fatty acids, although a study has shown an inverse association between EPA and CD risk.^115^ These data do not align with the results of this paper.

Preclinical studies document the impact of diet components associated with the risk of CD and UC on gut homeostasis. Fiber is a key component of healthy and Mediterranean diets. It is metabolized into short chain fatty acids (SCFA) by gut microbiota that widely regulate intestinal homeostasis, and include Bacteroides, Bifidobacterium, Blautia, Lachnospiraceae, Clostridia such as Faecalibacterium, Eubacterium, Roseburia, and Ruminococcus.^116^ SCFA directly modulate the activation and differentiation of immune cells, transduce signals in epithelial cells and increase intestinal IgA and systemic IgG.^116–118^ Ultra-processed foods are enriched in fat, sugar and salt but also contain emulsifiers, colors, nanoparticles and artificial sweeteners. Dietary questionnaires cannot differentiate the impact of food additives from that of macronutrients. Yet, preclinical studies have shown that food additives may contribute to the disruption of intestinal homeostasis.^119^ Emulsifiers have been studied extensively; they decrease bacterial diversity, increase pro-inflammatory bacteria such as adherent-invasive *Escherichia coli*, alter microbial gene regulation, decrease mucus thickness, increase gut permeability and activate inflammatory pathways.^120,121^

This study was based on a large number of participants and on validated questionnaires. The overall quality of studies included, as assessed by the STROBE criteria, was adequate. In the vast majority of the studies, diagnoses of CD and UC were certified by physicians or ICD codes. In addition, the prospective cohort study design reduced recall and reverse causality biases. The heterogeneity of most meta-analyses was low. This study also has limitations. First, most of the included studies were conducted in the USA and Europe. Second, most cohorts included middle to old age participants; our results might not be generalizable to younger people. Third, even if studies included are prospective, the assessment of diet using questionnaires may introduce a memory bias. There may also be a classification bias for UPF exposure as some items do not clearly fall into the NOVA classification. However, any classification error would be non-differential, and thus would underestimate potential associations. Fourth, definitions and scorings of Mediterranean diets differed between studies. Fifth, as in any epidemiological study, residual confounding bias is possible.

In conclusion, this paper adds further evidence and consistency to the role of diet in the risk of IBD, particularly CD. It could help to build prevention recommendations and clinical trials.

## Contributors

Antoine Meyer contributed to figures, study design, data collection and analysis, data interpretation, validation and writing.

Manasi Agrawal contributed to study design, supervision, data interpretation, writing, review and editing.

Einat Savin Shalom contributed data collection, data analysis, data interpretation, validation, original draft and writing.

Emily CL Wong contributed to data collection, writing.

Carrie Levinson contributed to methodology, literature search, study design, data collection, writing.

Stephanie Gold contributed to data interpretation, writing.

Neeraj Narula contributed to data collection and writing.

Jean-Frédéric Colombel contributed to study design, supervision, data analysis, data interpretation, writing.

Franck Carbonnel contributed to literature search, figures, study design, data collection, data analysis, data interpretation, validation, original draft, writing, review & editing.

All authors read and approved the final version of the manuscript.

Antoine Meyer, Einat Savin Shalom and Franck Carbonnel verified the underlying data.

## Data sharing statement

Data underlying the search results can be reproduced using the search protocol reported in the supplementary methods. Data underlying the meta-analyses is available on all corresponding forest plots. R codes for analyses are available upon request.

## Declaration of interests

Antoine Meyer: reports no conflicts of interest.

Manasi Agrawal: has received honoraria from Medscape.

Einat Savin Shalom: reports no conflict of interest.

Emily CL Wong: reports no conflict of interest.

Carrie Levinson: reports no conflicts of interest.

Stephanie Gold: Nestle Nutrition Institute Fellow 2023, Supported by a Crohn's and Colitis Foundation Career Development Award, Medical Board Members of Nutritional Therapy for IBD.

Neeraj Narula: holds a McMaster University AFP Clinician Researcher Award. Neeraj Narula has received honoraria from Janssen, Abbvie, Takeda, Pfizer, Sandoz, Novartis, Iterative Health, Innomar Strategies, Fresenius Kabi, Amgen, Organon, Eli Lilly, and Ferring.

Jean Frédéric Colombel: reports receiving research grants from AbbVie, Janssen Pharmaceuticals and Takeda; receiving payment for lectures from AbbVie, Amgen, Ferring Pharmaceuticals, Shire, and Takeda; receiving consulting fees from AbbVie, Amgen, Boehringer Ingelheim, Celgene Corporation, Celltrion, Enterome, Ferring Pharmaceuticals, Genentech, Janssen Pharmaceuticals, Eli Lilly, Medimmune, Merck, Novartis, Pfizer, Protagonist Therapeutics, Sandoz, Second Genome, Seres Therapeutics, Shire, Takeda, Theradiag and Theravance Biopharma; and hold stock options in Intestinal Biotech Development and Genfit.

Franck Carbonnel: reports receiving research grants from Alpha Wassermann, MaaT pharma, Mayoly Spindler, Medtronic, Nestlé; receiving payment for lectures from AbbVie, AlphaSigma, Ferring, Janssen, Eli Lilly, Nestlé Health Sciences, Pierre Fabre, Pileje, Takeda, Tillots, Viatris; receiving consulting fees from AbbVie, Janssen, MaaT pharma, Tillots.

## Appendix A Supplementary data

Supplementary data diet and risk of IBD revised2

STROBE statement
