# Supplementary material for: Impact of diet on inflammatory bowel disease risk: systematic review, meta-analyses and implications for prevention
Source: eClinicalMedicine. 2025 Jul 14;86:103353. doi: 10.1016/j.eclinm.2025.103353 (PMC12281061; doi:10.1016/j.eclinm.2025.103353)
Supplement: Supplementary data diet and risk of IBD revised2 [file mmc1.docx]

**Supplementary Methods & Results. Diet and risk of inflammatory bowel disease: systematic review and meta-analyses of prospective cohort studies**

**Literature search**

*Original searches run November 6, 2024; re-run on May 8, 2025 (highlighted sections are date filters added for the search re-run)*

*Ovid MEDLINE(R) Epub Ahead of Print and In-Process, In-Data-Review & Other Non-Indexed Citations and Daily <May 07, 2025>*

*1 exp Inflammatory Bowel Diseases/*

*2 Inflammatory Bowel Disease*.tw,kf.*

*3 ((ulcerative or ulcerous or mucosal or Idiopathic) adj3 (colitis or proctocolitis or colorectitis)).tw,kf.*

*4 (colitis adj3 (gravis or ulcerativa or ulcerosa)).tw,kf.*

*5 ((Crohn* or granulomatous) adj3 (disease* or enteritis or ileitis or ileitides or colitis or enterocolitis)).tw,kf.*

*6 ((regional or terminal) adj3 (enteritis or ileitis or ileitides or colitis or enterocolitis)).tw,kf.*

*7 (morbus adj3 crohn*).tw,kf.*

*8 1 or 2 or 3 or 4 or 5 or 6 or 7*

*9 "Diet, Food, and Nutrition"/ or exp Diet/ or exp Food/ or Diet Records/*

*10 (diet* or nutrition or nutritive or food* or drink* or beverage* or meal*).tw,kf.*

*11 9 or 10*

*12 cohort studies/ or prospective studies/*

*13 (cohort or prospective).mp.*

*14 (nested adj3 ("case control" or "case-control")).mp.*

*15 12 or 13 or 14*

*16 8 and 11 and 15*

*17 limit 16 to dt=20241106-20250508*

*18 limit 16 to rd=20241106-20250508*

*19 17 or 18*

*Embase Classic+Embase <1947 to 2025 May 07>*

*1 exp Inflammatory Bowel Disease/*

*2 Inflammatory Bowel Disease*.tw,kf.*

*3 ((ulcerative or ulcerous or mucosal or Idiopathic) adj3 (colitis or proctocolitis or colorectitis)).tw,kf.*

*4 (colitis adj3 (gravis or ulcerativa or ulcerosa)).tw,kf.*

*5 ((Crohn* or granulomatous) adj3 (disease* or enteritis or ileitis or ileitides or colitis or enterocolitis)).tw,kf.*

*6 ((regional or terminal) adj3 (enteritis or ileitis or ileitides or colitis or enterocolitis)).tw,kf.*

*7 (morbus adj3 crohn*).tw,kf.*

*8 1 or 2 or 3 or 4 or 5 or 6 or 7*

*9 exp diet/ or dietary intake/ or dietary pattern/ or food frequency questionnaire/ or exp food intake/ or nutrition/*

*10 (diet* or nutrition or nutritive or food* or drink* or beverage* or meal*).tw,kf.*

*11 9 or 10*

*12 cohort analysis/ or prospective study/*

*13 (cohort or prospective).mp.*

*14 (nested adj3 ("case control" or "case-control")).mp.*

*15 12 or 13 or 14*

*16 8 and 11 and 15*

*17 limit 16 to dd=20241106-20250508*

*18 limit 16 to rd=20241106-20250508*

*19 17 or 18*

*Scopus*

*( ( TITLE-ABS-KEY ( inflammatory AND bowel AND disease* ) OR TITLE-ABS-KEY ( ( ( ulcerative OR ulcerous OR mucosal OR idiopathic ) W/3 ( colitis OR proctocolitis OR colorectitis ) ) ) OR TITLE-ABS-KEY ( ( colitis W/3 ( gravis OR ulcerativa OR ulcerosa ) ) ) OR TITLE-ABS-KEY ( ( ( crohn* OR granulomatous ) W/3 ( disease* OR enteritis OR ileitis OR ileitides OR colitis OR enterocolitis ) ) ) OR TITLE-ABS-KEY ( ( ( regional OR terminal ) W/3 ( enteritis OR ileitis OR ileitides OR colitis OR enterocolitis ) ) ) OR TITLE-ABS-KEY ( ( morbus W/3 crohn* ) ) ) ) AND TITLE-ABS-KEY ( ( diet* OR nutrition OR nutritive OR food* OR drink* OR beverage* OR meal* ) ) AND TITLE-ABS-KEY ( ( cohort OR prospective OR ( nested W/3 ( "case control" OR "case-control" ) ) ) ) AND PUBYEAR > 2023 AND PUBYEAR < 2026*

**Supplementary figure 1**. Forest plot with studies reporting association between other food items and risks of Crohn’s disease. Results present highest quantile compared with lowest quantile. aHR, adjusted hazard ratio; CD, Crohn’s disease; CI, confidence interval.

**Supplementary figure 2**. Forest plot with studies reporting association between other food items and risks of ulcerative colitis. Results present highest quantile compared with lowest quantile. aHR, adjusted hazard ratio; UC, ulcerative colitis; CI, confidence interval.

**Supplementary figure 3**. Forest plot for sensitivity analyses where exposure had the same categorization, for risks of Crohn’s disease. Results present highest quantile compared with lowest quantile. aHR, adjusted hazard ratio; CD, Crohn’s disease; CI, confidence interval.

**Supplementary figure 4**. Forest plot for sensitivity analyses where exposure had the same categorization, for risks of ulcerative colitis. Results present highest quantile compared with lowest quantile. aHR, adjusted hazard ratio; UC, ulcerative colitis; CI, confidence interval.

| **Supplementary table 1. Characteristics of included studies** | | | | | | | | | |
| --- | --- | --- | --- | --- | --- | --- | --- | --- | --- |
| **Author, year^reference^** | **Database and Country** | **Participants** | **Female gender**  **(%)** | **Mean age at recruitment (years)** | **Mean follow-up (years)** | **Design** | **CD/UC**  **Cases**  **(N)** | **Method of dietary assessment** | **Variables included within fully adjusted analyses** |
| Agrawal, 2024^81^ | Danish National Birth Cohort, Norwegian Mother, Father, and Child Cohort, and All Babies in Southeast Sweden | 169,510 | NA | At birth | CD 16.3  UC 22.3 | cohort | 301/230 | Specific questionnaire | sex, maternal IBD status, paternal IBD status, maternal education level, mode of delivery, maternal smoking during pregnancy, maternal age at delivery, preterm birth, small for gestational age |
| Ananthakrishnan, 2012^51^ | NHS: USA | 72,719 | 100 | 53 | 20.7 | cohort | 122/123 | Validated FFQ | Smoking, Oral contraceptive, postmenopausal hormone use, BMI, physical activity |
| Ananthakrishnan, 2013^35^ | NHS: USA | 170,776 | 100 | 43 | 19.4 | cohort | 269/338 | Validated FFQ | Smoking, oral contraceptive, postmeNpausal hormone, BMI, aspirin, NSAIDs, menopausal status |
| Ananthakrishnan, 2014^41^ | NHS: USA | 170,805 | 100 | 43 | 19.4 | cohort | 269/338 | Validated FFQ | Smoking, oral contraceptive, postmeNpausal hormone, BMI, aspirin, NSAIDs |
| Ananthakrishnan, 2015^44^ | NHS/NHS2: USA | 170,809 | 100 | 42.9 | 19.4 | cohort | 269/338 | Validated FFQ | smoking, menopausal hormone therapy, oral contraceptives,NSAIDs, diet fibre and vitamin D, Physical activity BMI |
| Andersen, 2018^36^ | EPIC: France, Italy, UK, Netherlands, Germany, Sweden, Denmark, Greece | 401,326 | CD 73.1  UC 58.4 | CD 49.6  UC 51.6 | CD 5.3  UC  4.8 | Nested Case control | 104/221 | Validated FFQ | Smoking, energy intake |
| Anneberg, 2025^84^ (abstract) | Danish National Birth Cohort | 63,877 | NA | newborns | NA | cohort | NA | FFQ | parental IBD diagnosis, maternal educational level, gestational weight gain, smok  ing, energy intake, nutritional supplement use, antibiotics use. |
| Bergmann, 2017^54^ | EPIC: France, Italy, UK, Netherlands, Germany, Denmark | 262,451 | CD 80.1  UC 62.8 | CD 50.8  UC 52.8 | 3.5 | Nested case control | 84/198 | Validated FFQ | Smoking, education |
| Casey, 2022^55^ | NHS/NHS2/ HPFS: USA | 237,835 | 79 | 46.4 | 21.7 | cohort | 370/486 | Validated FFQ | age, BMI, sex, physical activity, smoking, AHEI, NSAID, family history of IBD |
| Chan, 2014 (IBD)^32^ | EPIC: France, Italy, Greece, UK, Netherlands, Germany, Sweden, Denmark | 401,326 | CD 72.7  UC 57.3 | CD 50.1  UC 51.7 | 4.4 | Nested case control | 110/244 | Validated FFQ | total energy intake, BMI, smoking, metabolic rate, physical activity |
| Chan, 2014 (APT)^40^ | EPIC: UK, Netherlands, Germany, Sweden, Denmark | 229,702 | 64 | 56.4 | 5.4 | Nested Case control | 73/0 | Validated FFQ | Smoking, dietary energy, dietary vitamin D, other dietary fatty acids |
| Chen, 2023^74^ | UK Biobank | 185,849 | 54.8 | 56.2 | 9.8 | cohort | 251/590 | web-based 24h dietary assessment | sex, ethnicity, education, TDI, polygenic risk scores, smoking, alcohol, physical activity, body, BMI, CRP, urine sodium,nutrient intake, energy, alternative healthy eating index [AHEI], Charlson Comorbidity Index, family history of bowel cancer, IBD-related drugs |
| Chen 2025 (CGH)^48^ | UK Biobank | 186,195 | 55.2 | 56.3 | 11.4 | cohort | 396/809 | Web-based 24-h dietary recall | age, sex, ethnicity, TDI, education, smoking (never smoked, alcohol consumption, BMI, physical activity, CRP,  baseline comorbidities,  use of antioxidant  supplements. |
| Chen, 2025 (Lancet Reg Heal - Eur)^73^ | UK Biobank | 187,888 | 55 | 50.7 | 11.6 | cohort | 286/639 | Web-based 24-h dietary recall | age, sex, TDI, education, ethnicity, BMI, smoking status, alcohol consumption, exercise time, total energy intake,  total sugar intake, intake of ultra-processed  food. CRP, IBD-related medication. |
|  | EPIC:  Denmark, France, Germany, Greece, Italy, Netherlands, Sweden, UK. | 341,539 | 69.9 | 52.1 | 14.5 | cohort | 156/ 392 | Validated FFQ | age, sex, education, ethnicity, BMI, smoking status, alcohol consumption, exercise time, total energy intake,  total sugar intake, intake of ultra-processed  food. CRP, IBD-related medication. |
| Daher, 2025^72^ (abstract) | Israeli IDF database | 1,049,005 | 40 | NA | 2.8 | Nested case-control | 1427/612 | questionnaire | age, sex, BMI, country of origin, general intelligence tests, socioeconomic status. |
| Deng, 2023^30^ | UK Biobank | 470,669 | 54.7 | 56.5 | 12.1 | cohort | 543/939 | Validated FFQ | age, sex, ethnicity, educational level, TDI, smoking,alcohol, BMI, physical activity, Charlson Comorbidity Index, major food groups |
| De Freitas, 2025^85^ (abstract) | Danish National Birth Cohort | 67,770 | NA | newborns | NA | cohort | 106/86 | FFQ | maternal educational level, gestational weight gain, alcohol intake, smoking, antibiotics, parental IBD, diet quality (Healthy Eating Index), offspring antibiotic consumption. |
| De Silva 2014^39^ | EPIC Norfolk UK | 25,639 | 42.3 | 64.9 | 3.8 | Nested case control | 0/26 | 7-day food diary | Total energy intake, smoking, linoleic acid, EPA, DHA, a-linolenic acid, oleic acid, fish oil supplements, aspirin, social class |
| Dong, 2022^27^ | EPIC: France, Italy, Spain, UK, Netherlands, Germany, Sweden, Denmark | 413,593 | 69 | 52.5 | 16.8 | cohort | 177/418 | Validated FFQ | Smoking, Non-protein energy, educational level, physical activity, BMI |
| Fu, 2022 (APT)^37^ | UK Biobank | 121,490 | 55.7 | 56.2 | 10.2 | cohort | 143/367 | web-based 24h dietary assessment | Age, sex, ethnicity TDI, education, Smoking, alcohol, physical activity, BMI, total sugar, energy intake diet quality score (AHEI, Med score) |
| Fu, 2022 (Nutrients)^25^ | UK Biobank | 482,887 | 54.4 | 56.6 | 12.1 | cohort | 851/1866 | Validated FFQ | age, sex, ethnicity, TDI, physical activity, smoking, alcohol, BMI, NSAIDS, PPI, antibiotics, CRP, Charlson |
| Fu, 2024  (IBD)^28^ | UK biobank | 265,839 | 55.3 | 57.2 | 11.8 | cohort | 501/1053 | Validated FFQ | age, sex, ethnicity, educational level, employment status, income, BMI, Smoking, alcohol, physical activity, aspirin NSAIDs, TSI |
| Guevara, 2021^68^ | EPIC-Spain | 32,663 | 61.7 | 48.9 | 20.7 | cohort | 32/57 | Validated FFQ | Smoking education, level, and physical activity, BMI |
| Guo, 2024 (IBD)^80^ | All Babies in  Southeast Sweden and The Norwegian Mother, Father  and Child Cohort Study | 81,272 | 48 | 1 | 16.2 | cohort | 131 CD  97 UC  79 IBDU | unvalidated FFQ | Maternal : origin, maternal, age, immune-mediated comorbidities, smoking in pregnancy, delivery mode. Child: sex, birth weight, gestational age, full breastfeeding duration, antibiotic use by 1 year of age, HEI at 1 year |
| Guo, 2024 (Gut)^83^ | All Babies in  Southeast Sweden and The Norwegian Mother, Father  and Child Cohort Study | 81,280 | 48 | 1 | 16.2 | cohort | 131 CD  97 UC  79 IBDU | unvalidated FFQ | Maternal : origin, maternal, age, immune-mediated comorbidities, smoking in pregnancy, delivery mode. Child: sex, birth weight, gestational age, full breastfeeding duration, antibiotic use by 1 year of age, HEI at 1 year |
| Guo, 2024 (Am J Clin Nutr)^79^ | Norwegian Mother, Father  and Child Cohort Study | 85,129 | 48.8 | newborns | 16.1 | cohort | CD 119  UC 76  IBDU 73 | Validated FFQ in mothers | Sex, parental IBD, ethnicity, education, comorbidities, BMI of the mother |
| Hammer, 2019^24^ | Faroe Islands | 5,698 | NA | NA | NA | cohort | 5/32 | Self-reported questionnaire  unvalidated | No |
| Hart, 2008^33^ | EPIC: UK, Sweden, Denmark, Germany, Italy | 260,686 | 51.1 | 58.8 | 3.8 | Nested case control | 0/139 | Validated FFQ | Age, sex, physical activity, smoking, drinking habits, medication |
| Hart, 2009^38^ | EPIC: Italy, UK, Germany, Sweden, Denmark | 203,193 | 47 | 60 | 4 | Nested Case control | 0/126 | Validated FFQ | age, total energy intake, smoking and centre. individual fatty acids, aspirin, educational level |
| Huang, 2022^43^ | UK biobank | 447,890 | 53.5 | 56.2 | 8 | cohort | 533/1,185 | Validated FFQ | Age, sex, ethnicity, assessment centers, BMI, education, TDI, income, smoking status, alcohol, physical activity, other diet supplements, medication, dietary intake, MET, use of NSAIDSs/hormones and missing variables. |
| Jantchou, 2010^16^ | EPIC France | 67,581 | 100 | 52.8 | 10.4 | cohort | 30/43 | Validated FFQ | BMI, alcohol, physical activity, oral contraceptives, menopause hormonal treatment, smoking, level of education |
| Jantchou, 2014^52^ | EPIC France | 91,879 | 100 | 49.3 | 13.1 | cohort | 30/43 | Validated FFQ | BMI, physical activity smoking, menopausal hormone therapy, education, skin complexion (fair vs dark) |
| Jiang, 2024^57^ | UK biobank | 121,978 | 55.7 | 56.1 | 13.7 | cohort | 192/478 | web-based 24h dietary assessment | ethnicity, education, TDI, BMI, smoking, alcohol, physical activity, healthy diet score, aspirin, Charlson Comorbidity Index, PRS, and the first 5 genetic principal components |
| John, 2010^42^ | EPIC: Norfolk UK | 25,639 | 45.5 | 66.7 | 4.2 | Nested Case control | 0/22 | 7-day  food diary | Smoking, total energy intake, liNleic acid, oleic acid.  Odds ratios for EPA and DHA, but Nt total n-3  PUFA intake adjusted for the dietary intake of alpha-liNlenic acid |
| Khalili, 2014  (Abstract)^78^ | NHS/NHS2: USA | 181,843 | 100 | NA | NA | cohort | 256/318 | Validated FFQ | age, smoking, BMI, physical activity, history of appendectomy, use of oral contraceptives, menopausal hormone therapy, NSAIDs |
| Khalili, 2016^46^ | NHS/NHS2: USA | 194,711 | 100 | 43.2 | 16.5 | cohort | 273/335 | Validated FFQ | body weight, smoking, NSAIDs, menopausal hormone therapy, oral contraceptives, physical activity, latitude of residence at age 30, appendectomy, ancestry |
| Khalili, 2017^15^ | NHS/NHS2: USA | 165,331 | 100 | 42-44 | 18.4 | cohort | 261/321 | Validated FFQ | ethnicity, physical activity, body weight, smoking, latitude of residence, NSAIDs, meNpausal hormone therapy, oral contraceptive |
| Khalili, 2019^31^ | Swedish Mammography + Swedish Men | 83,042 | 45.8 | 60 | 11 | cohort | 143/349 | Validated FFQ | Sex, age, BMI, smoking, total  caloric, protein, fiber intakes, NSAIDs, physical activity |
| Khalili, 2020^26^ | Swedish Mammography + Swedish Men | 83,147 | 45.9 | 61 | 17 | cohort | 164/395 | Validated FFQ | Sex, age, BMI, education level, smoking, total caloric intake,physical activity (Met-­ hr/week). |
| Khalili, 2023^63^ | Swedish Mammography + Swedish Men | 83,147 | 46 | 61 | 16.9 | cohort | 164/395 | Validated FFQ | Age, BMI, smoking, physical activity, energy intake, education, cohort |
| Kim, 2023 (Abstract)^60^ | NHS/NHS2/ HPFS: USA | 218,509 | NA | NA | NA | cohort | 385/516 | Validated FFQ | age, sex, BMI, family history of IBD |
| Liu, 2022^56^ | UK biobank | 502,505 | 55.5 | 69.5 | NS | cohort | 7095 | specific questionnaire | age, sex, ethnicity education level, BMI, current employment status, TDI, overall health rating, smoking status, usually walking pace, comorbidities, sleep phenotype |
| Lo, 2020^67^ | NHS/NHS2/ HPFS: USA | 208,834 | 80 | 44.6 | 23.7 | cohort | 328/428 | Validated FFQ | race, smoking, BMI, physical activity, contraceptives, hormone replacement therapy. Energy (residuals), fiber |
| Lo, 2022^75^ | NHS/NHS2: USA | 245,112 | 83.0 | 45 | 22.3 | cohort | 369/488 | Validated FFQ | ethnicity, family history of IBD, smoking, BMI, physical activity, energy, AHEI-2010, NSAIDs, oral contraceptives, menopausal hormone therapy |
| Lopes, 2022^58^ | NHS/NHS2/ HPFS: USA | 208,280 | 80 | 45.3 | 25.3 | cohort | 337/447 | Validated FFQ | BMI, physical activity, smoking, NSAID, oral contraceptives, menopausal hormone therapy, appendectomy, family history of IBD, energy (residuals), AHEI. |
| Lopes, 2023^19^ | Primary cohort: NHS/NHS2/ HPFS: USA  Validation cohorts: Swedish Mammography + Swedish Men + EPIC-Europe | 208,070 (primary cohort)  482,229 (validation cohorts) | 75-85  (primarycohort) | 42.9-47.7 (primary cohort) | 24.6 | cohort | 346/456 | Validated FFQ | age, time period, cohort, appendectomy, family history of IBD |
| Lopes, 2025^34^ | NHS/NHS2/ HPFS: USA | 223,283 | NA | NA | 24.4 | cohort | 371/481 | Validated FFQ | BMI, IBD-related medication, history of appendectomy, smoking status, physical activity, family history of IBD,  alternate healthy eating index (AHEI), total daily caloric  intake. |
| Lu, 2017^47^ | EPIC: France, Italy, UK, Netherlands, Germany, Sweden, Denmark, Greece | 401,326 | CD 72.7  UC 57.4 | CD 50.1  UC 51.7 | CD 5.5  UC 5.8 | Nested Case control | 110/244 | Validated FFQ | educational level, smoking, total energy. |
| Lu, 2024^49^ | UK biobank | 187,709 | 55.0 | 59.04 | 9.7 | cohort | 256/607 | web-based 24h dietary assessment | age, sex, race, education level, smoking status, physical activity level, BMI, TDI, comorbidities (CCI index), CRP level, NSAIDs, PPI, antibiotics, OCP, Dietary variables. |
| Meyer, 2023^76^ | EPIC: France, Italy, UK, Netherlands, Germany, Sweden, Denmark, Spain | 413,590 | 68.6 | 51.7 | 13.2 | cohort | 179/431 | Validated FFQ | Center, age, sex, smoking, BMI, physical activity, energy, education level, alcohol |
| Meyer, 2024^18^ | EPIC: France, Italy, UK, Netherlands, Germany, Sweden, Denmark, Spain | 394,255 | 68.1 | 52.1 | 13.6 | Prospective cohort | 184/459 | Validated FFQ | Centre, sex, smoking, BMI, physical activity, educational level, energy, alcohol |
| Meyer, 2025^14^ | EPIC: France, Italy, UK, Netherlands, Germany, Sweden, Denmark, Spain | 394,255 | 68.1 | 52.1 | 13.6 | cohort | 184/459 | validated FFQ | Center, sex, smoking, BMI, physical activity, educational level, energy, alcohol |
| Narula, 2021 (BMJ)^29^ | 21 low, middle, and high income countries across  seven geographical regions (Europe and North  America, South America, Africa, Middle East, south  Asia, South East Asia, and China) | 116,087 | 59.2 | 50.2 | 9.7 | cohort | 90/377 | Validated FFQ | Age, sex, Geographical region, education, alcohol intake, smoking status, physical activity, energy intake, BMI, waist to hip ratio, urban vs rural location |
| Narula, 2021 (Gastroenterology)^66^ | 7 countries (Argentina,  Brazil, Canada, Chile, Poland, South Africa, and Sweden) | 28,428 | NA | NA | 10.1 | cohort | 49/134 | Validated FFQ | age, sex, household income, education, alcohol, smoking, energy intake, BMI, waist-to-hip  ratio, physical activity, NSAIDs, oral contraceptive use, urban or rural |
| Nguyen, 2020^59^ | Health Professionals Follow-up Study (HPFS): USA | 219,701 | NA | NA | NA | cohort | 376/506 | Validated FFQ | age, caloric intake, smoking, body mass index, physical activity, family history of IBD, NSAID use, OCP/menopausal hormone use |
| Opstelten, 2016^22^ | EPIC: France, Italy, Greece, UK, Netherlands, Germany, Sweden, Denmark | 401,326 | CD 72.7  UC 57.4 | CD 50.1  UC 51.7 | CD 4.8  UC 5.1 | Nested case-control | 110/244 | Validated FFQ | gender, age and date at recruitment, center, energy, smoking, metabolic rate, physical activity |
| Opstelten, 2018^53^ | EPIC: France, Italy, UK, Netherlands, Germany, Sweden, Denmark, Greece | 359,728 | CD 77.8  UC 48.5 | CD 48.8  UC 51.6 | CD 4.7  UC 4.1 | Nested Case control | 72/169 | Validated FFQ | Smoking,PTH, linoleic acid, docosahexaenoic acid, BMI, physical activity, alcohol, total dairy, products, dietary calcium, season/month |
| Peters, 2022^10^ | LifeLines: The Netherlands | 125,445 | 58.5 | 44.8 | NA | Nested Case control | 97/224 | Validated FFQ | age, gender, body mass index, and smoking status |
| Racine, 2016^62^ | EPIC: France, Italy, UK, Netherlands, Germany, Sweden, Denmark | 366,351 | CD 73  UC 61 | CD 50.3  UC 51.5 | CD 3.8  UC 4.6 | Nested Case control | 117/256 | Validated FFQ | Smoking, BMI, dietary energy |
| Rubin, 2020^17^ | DCH: Danish Diet, Cancer  and Health cohort | 56,075 | 48 | 56.2 | 20.0 | cohort | 118/445 | Validated FFQ | age, sex, energy, alcohol, smoking, education comorbidity,civil status |
| Sasson 2024^20^ (abstract) | NHS/NHS2/ HPFS: USA | 197,765 | NS | NA | 25 | cohort | 347/428 | Validated FFQ | BMI, physical activity, NSAIDs, family history, AHEI, cohort, smoking |
| Sauk, 2017^61^ (Abstract) | NHS/NHS2: USA | 165,335 | 100 | NA | NA | cohort | 261/321 | Validated FFQ | NA |
| Song, 2024^21^ | China Kadoorie Biobank | 456,590 | 58.9 | 51.8 | 12.1 | cohort | 0/312 | Unvalidated FFQ | sex, region, education, alcohol, smoking, physical activity, BMI, income, marital status, energy intake, hygiene, oral contraceptives, comorbidities, antibiotics, NSAIDs |
| Sun, 2022^70^ | UK biobank | 430,384 | 54.5 | 56.7 | 12 | cohort | 707/1576 | Validated FFQ | age, age-square, sex, education, Townsend deprivation index, Charlson comorbidities index, and first 20 principal components of ancestry |
| Sun, 2024^50^ | UK Biobank | 188,044 | 55.2 | 59 | 9.7 | cohort | 255/606 | web-based 24h dietary assessment | age, sex, race, education level, physical activity level, BMI, use of medication (anti-inflammatory drugs, proton pump inhibitors, and antibiotics) |
| Thompson, 2000^82^ | National Survey of Health &  Development and the National Child  Development Study: UK | 11,407 | NA | newborns | NA | Nested case-control | 26/29 | Survey | Sex, social class |
| Vasseur, 2020^45^ | Nutrinet: France | 105,832 | 78 | 43.3 | 2.3 | cohort | 27/48 | Validated FFQ | Age, sex, income, education level, marital status, residence, BMI, physical activity, smoking, hormonal contraception, number of 24-hour dietary records, energy intake. |
| Vasseur, 2021^77^ | Nutrinet: France | 105,832 | 78 | 43.3 | 2.3 | cohort | 27/48 | Validated FFQ | Age, sex, income, education level, marital status, residence, BMI, physical activity, smoking, hormonal contraception, number of 24-hour dietary records, energy intake. |
| Xia, 2024^64^ | UK Biobank | 197,391 | NA | NA | 11.1 | cohort | 260/601 | Web-based 24-h dietary recall | age, sex, ethnicity, smoking status, alcohol consumption, daily sleeping time, BMI, physical activity, medication intake, comorbidities, index of multiple deprivation (IMD), self-reported longstanding illness. |
| Yang, 2024^69^ | UK biobank | 260,836 | 51.9 | 57 | 12.3 | cohort | 502/1070 | Validated FFQ | age, sex, education, alcohol, TDI, depression |
| Ye, 2024^23^ | UK biobank | 187,490 | 55.0 | 56.2 | 10.7 | cohort | 250/575 | web-based 24h dietary assessment | Age, sex, ethnicity, education, social deprivation, smoking, physical activity, BMI, total energy/sugar intake, comorbidities, cancer history, depression symptoms. |
| Ye, 2025^71^ | UK Biobank | 187,558 | 55 | 56 | 13.15 | cohort | 571/1066 | Web-based 24-h dietary recall | age, sex, race, TDI, smoking status, alcohol intake, physical activity, sleep duration, BMI. |
| AHEI: Alternate Healthy Eating Index, BMI: body mass index, CD: Crohn’s disease, EPIC: European Prospective Investigation into Cancer and Nutrition, FFQ: food frequency questionnaire, HPFS : Health Professionals Follow-up Study, IBD: inflammatory bowel disease, IBDU: inflammatory bowel disease-unclassified, NA: not available, NCDS: National child development study, NHS: Nurses' Health Study, NSAIDs: nonsteroidal anti-inflammatory drug, NSHD: National Survey of Health and Development, UC: ulcerative colitis, TDI: Townsend deprivation index | | | | | | | | | |

| **Supplementary table 2. Demographic characteristics of largest sample size or most recent articles of adult cohort studies** | | | | | | | | |
| --- | --- | --- | --- | --- | --- | --- | --- | --- |
| **Author, year** | **Database and Country** | **Participants** | **% of total participants** | **Female (%)** | **Mean age at recruitment** | **Mean follow-up (years)** | **CD cases** | **UC cases** |
| Meyer, 2025^14^ | EPIC | 394 255 | 19,3% | 68,1% | 52,1 | 13,6 | 184 | 459 |
| Fu, 2022^25^ | UK biobank | 482 887 | 23,6% | 54,4% | 56,6 | 12,1 | 851 | 1866 |
| Khalili, 2023^63^ | Swedish Mammo | 83 147 | 4,1% | 45,8% | 61 | 16,9 | 164 | 395 |
| Lopes, 2025^34^ | NHS/NHS2/HP: USA | 223 283 | 10,9% | 82,0% | 57 | 24,5 | 371 | 481 |
| Narula, 2021 (BMJ)^29^ | PURE | 116 087 | 5,7% | 59,2% | 50,2 | 9,7 | 90 | 377 |
| Peters, 2022^10^ | Netherlands | 125 445 | 6,1% | 58,5% | 44,8 |  | 97 | 224 |
| Rubin, 2020^17^ | DCH | 56 075 | 2,7% | 48,0% | 56,2 | 20 | 118 | 455 |
| Vasseur, 2020^45^ | Nutrinet: France | 105 832 | 5,2% | 78,0% | 43,3 | 2,3 | 27 | 48 |
| Song, 2024^21^ | China | 456 590 | 22,3% | 58,9% | 51,8 | 12,1 | 0 | 312 |
| Total |  | 2 043 601 | 100% | 62,3% | 53,1 | 12,8 | 1902 | 4617 |
| *Sex, age, and follow-up totals were weighted for the number of participants. CD: Crohn's disease; UC: ulcerative colitis.* | | | | | | | | |

| **Supplementary table 3. Results of included studies for protein exposure** | | | | | |
| --- | --- | --- | --- | --- | --- |
| **Author (year)^reference^** | **Exposure** | **Measure** | **Dietary analyses** | **Risk measure** | **P trend** |
| Dong, 2022^27^ | Total protein   - CD - UC | aHR | Quartiles | 4^th^ vs 1^st^ quartile  1.43 (0.76 – 2.70)  1.18 (0.78-1.77) | 0.38  0.58 |
|  | Animal protein   - CD - UC |  |  | 1.05 (0.60-1.83)  0.96 (0.67-1.39) | 0.70  0.69 |
|  | Vegetable protein   - CD - UC |  |  | 0.81 (0.45-1.47)  1.18 (0.80-1.72) | 0.67  0.49 |
|  | Meat   - CD - **UC** |  |  | 1.28 (0.76-2.16)  **1.40 (0.99-1.98)** | 0.11  **0.01** |
|  | **Red meat**   - CD - **UC** |  |  | 1.08 (0.63-1.84)  **1.61 (1.10-2.36)** | 0.37  **0.007** |
|  | Processed meat   - CD - UC |  |  | 1.19 (0.71-1.98)  1.18 (0.84-1.65) | 0.39  0.29 |
|  | Fish   - CD - UC |  |  | 0.87 (0.54-1.40)  0.95 (0.68-1.32) | 0.90  0.88 |
|  | Egg   - CD - UC |  |  | 1.07 (0.65-1.78)  0.93 (0.67-1.30) | 0.99  0.98 |
|  | Dairy products   - CD - UC |  |  | 0.84 (0.54-1.30)  0.88 (0.65-1.19) | 0.53  0.46 |
|  | Poultry   - CD - UC |  |  | 1.42 (0.87-2.34)  0.92 (0.67-1.26) | 0.33  0.99 |
| Fu, 2022 (Nutrients)^25^ | Fish   - IBD   Processed meat   - IBD   Unprocessed meat   - IBD | aHR | Quintiles | 0.99 (0.91, 1.07)   1. (0.92, 1.09)   1.04 (0.94, 1.16) | 0.744  0.943  0.413 |
| Fu, 2024^28^ | Oily fish  -IBD  -CD  -UC  Nonoily fish  -IBD  -CD  -UC | aHR | Quartiles | 0.88 (0.73-1.06)  **0.65 (0.47-0.89)**  1.03 (0.81-1.30)  0.97 (0.75-1.25)  0.71 (0.47-1.08)  1.13 (0.82-1.56) | 0.08  **0.002**  0.86  0.94  0.19  0.47 |
| Hammer, 2019^24^ | Fish  UC  Whale  UC | Rate ratio | Yes/no or low/high | 1.24 (0.37–4.14)  0.86 (0.39–1.87) |  |
| Jantchou, 2010^16^ | **Total protein**   - **IBD** - **CD** - UC   **Animal protein**   - **IBD** - CD - **UC**   Vegetable protein   - IBD - CD - UC   **Meat IBD**  **Fish IBD**  Eggs IBD  Dairy products IBD | **HR** | **Tertiles** | **3^rd^ vs 1^st^ tertile**  **3.31 (1.41 – 7.77)**  **3.34 (0.90 – 12.4)**  3.24 (1.07 – 9.84)  **3.03 (1.45-6.34)**  2.70 (0.69-10.52)  **3.29 (1.34-8.04)**  1.31 (0.519-2.88)  1.04 (0.28-3.80)  1.70 (0.59-4.81)  **1.87 (1.00 – 3.49)**  **1.83 (1.00 – 3.36)**  0.97 (0.52-1.78)  0.94 (0.53-1.67) | **0.007**  **0.04**  0.06  **0.005**  0.33  **0.005**  0.44  0.98  0.33  **0.02**  **0.05**  0.91  0.93 |
| Khalili, 2017^15^ | Red meat   - CD - UC   Processed meat   - CD - UC | aHR | Quintiles | 0.68 (0.44–1.05)  1.10 (0.72–1.65)  0.99 (0.64–1.54)  1.12 (0.77–1.62) | 0.40  0.08  0.99  0.86 |
| Khalili, 2020^26^ | Red and processed meat   - CD - UC   Fish   - CD - UC   Fermented dairy products   - CD - UC | aHR | Quartiles | 1.02 (0.71-1.48)  1.08 (0.85-1.39)  0.97 (0.68-1.28)  1.13 (0.92-1.39)  0.82 (0.59-1.12)  1.05 (0.85-1.30) | NS  NS  NS  NS  NS  NS |
| Lopes, 2023^19^ | Red meat  CD  UC | aHR | Quintiles | 0.92 (0.63-1.33)  0.96 (0.70-1.31) | 0.74  0.73 |
| Meyer, 2024^18^ | Total protein   - CD - UC | aHR | Quartiles | 1.49 (0.76-2.91)  1.21 (0.79-1.84) |  |
| Narula, 2021 (BMJ)^29^ | White meat   - IBD - CD - UC   **Processed meat**   - **IBD** - CD - **UC**   Red meat   - IBD - CD - UC   Dairy   - IBD - CD - UC | aHR | Tertiles | 1.38 (0.97-1.95)  **2.07 (1.14-3.76)**  2.50 (0.39-16.03)  **2.19 (1.16-4.16)**  1.12 (0.81-1.53)  1.26 (0.86-1.83) | 0.10  **0.01**  0.72  0.49 |
| Opstelten, 2016^22^ | Total dairy products   - CD - UC   Milk   - CD - UC   Yogurt   - CD - UC   Cheese   - CD - UC   Calcium   - CD - UC | aOR | Quartiles | 0.61 (0.32-1.19)  0.80 (0.50–1.30)  0.61 (0.33-1.16)  0.81 (0.51–1.29)  0.78 (0.40-1.52)  0.95 (0.60-1.48)  0.85 (0.42-1.72)  0.83 (0.52-1.32)  0.63 (0.28-1.42)  0.81 (0.49-1.34) | 0.19  0.40  0.36  0.32  0.62  0.74  0.64  0.60  0.23  0.60 |
| Rubin, 2020^17^ | Meat   - IBD |  |  | 0.95 (0.75-1.21) | NS |
| Sasson 2024^20^ (abstract) | Dairy intake  CD  UC  UC latency analysis  dairy  yogurt | aHR | Quintiles | 1.09 (0.77-1.55)  0.84 (0.62-1.12)  0.71 (0.52-0.96)  0.68 (0.48-0.96 | 0.822  0.128  0.017  0.028 |
| Song, 2024^21^ | **Egg UC** | **aHR** | **Quintiles** | **2.29 (1.26–4.16)** | **P<0.001** |
| Ye, 2024^23^ | Red meat  CD  UC  Fish  CD  UC | aHR | Tertiles | 1.02 (0.77-1.34)  0.92 (0.77-1.10)  0.79 (0.60-1.03)  0.92 (0.77-1.10) |  |

| **Supplementary table 4. Results of included studies for carbohydrates exposure** | | | | | |
| --- | --- | --- | --- | --- | --- |
| **Author (year)^reference^** | **Exposure** | **Measure** | **Dietary analyses** | **Risk measure** | **P trend** |
| Ananthakrishnan, 2013^35^ | Fiber   - CD - UC   Fiber from fruits   - **CD** - UC   Fiber from vegetables   - CD - UC   F from cruciferous   - CD - UC   F from Cereals   - CD - UC   F from Whole grain   - CD - Uc   F from Bran   - CD - UC   F from Legumes   - CD - UC | aHR | Quintiles | 0.59 (0.39-0.90)  0.82 (0.58-1.17)  **0.57 (0.38-0.85)**  0.78 (0.54-1.12)  0.74 (0.50-1.07)  0.88 (0.61-1.25)  0.78 (0.54-1.13)  0.95 (0.67-1.36)  0.85 (0.57-1.26)  1.26 (0.88-1.81)  1.07 (0.71-1.60)  1.27 (0.88-1.83)  0.85 (0.56-1.28)  1.13 (0.79-1.63)  0.98 (0.66-1.44)  1.23 (0.87-1.72) | 0.08  0.41  **0.02**  0.15  0.25  0.35  0.35  0.64)  0.95  0.46  0.79  0.42  0.65  0.97  0.88  0.21 |
| Andersen, 2018^36^ | Fiber   - CD - UC   Fiber from fruits   - CD - UC   Fiber from vegetables   - CD - UC   F from Cereals   - CD - UC | OR | Quartiles | 0.83 [0.38 1.81] 1.22 (0.71-2.08)  0.84 (0.26-2.70)  1.08 (0.67-1.77)  0.84 (0.21-3.39)  0.70 (0.41-1.18)  1.13 (0.39-3.32)  1.09 (0.64-1.99) |  |
| Chan, 2014 (IBD)^32^ | Total carbohydrates   - CD - UC   Total sugar   - CD - UC   Starch   - CD - UC | aOR | Quintiles | 0.87 (0.24–3.12)  1.46 (0.62-3.46)  0.76 (0.28-2.08)  1.12 (0.57-2.17)  0.74 (0.23-2.40)  1.73 (0.83-3.62) | 0.70  0.41  0.50  0.71  0.69  0.17 |
| Deng, 2023^30^ | Fiber  - **CD**  - UC  Fruit fiber   - **CD** - UC   Vegetable fiber   - CD - UC   Bread fiber   - **CD** - UC   Cereal fiber   - CD - **UC** | aHR | Quintiles | **0.48 (0.32-0.72)**  0.92 (0.69-1.24)  **0.79 (0.64-0.98)**  1.18 (0.90-1.54)  0.88 (0.60-1.30)  1.01 (0.76-1.35)  **0.75 (0.57-0.98)**  1.06 (0.87-1.30)  0.92 (0.69-1.23)  **0.79 (0.64-0.98)** | **0.013**  0.327  **0.014**  0.205  0.149  0.836  **0.021**  0.984  0.449  **0.01** |
| Fu, 2022 (Nutrients)^25^ | **Fruits**   - **IBD**   **Vegetables**   - **IBD**   Whole grains   - IBD   Refined grains   - IBD | aHR | Quintiles | **0.90 (0.83-0.98)**  **0.88 (0.80, 0.97)**  0.89 (0.79, 1.01)  0.96 (0.87, 1.06) | **0.01**  **0.013**  0.064  0.376 |
| Fu, 2022 (APT)^37^ | Sugar-sweetened beverages   - CD - UC   Artificially-sweetened bev   - **CD** - UC   Natural juices   - CD - UC | aHR | Tertiles | 2.05 (1.22-3.46)  1.31 (0.89-1.92)  **0.42 (0.15-1.15)**  1.05 (0.67-1.65)  1.24 (0.67-2.28)  1.01 (0.66-1.55) | 0.181  0.432  **0.032**  0.281  0.507  0.448 |
| Hart, 2008^33^ | Fiber (g/day)  UC | OR | Quartiles | 1.03 (0.84–1.25) | 0.8 |
| Jantchou, 2010^16^ | Total carbohydrates   - IBD - CD - UC | HR | Tertiles | 3^rd^ vs 1^st^ tertile  0.68 (0.37 – 1.27)  1.31 (0.42 – 4.14)  0.51 (0.24 – 1.08) | 0.26  0.46  0.12 |
| Khalili, 2019^31^ | Sweetened beverage   - CD - UC | aHR | Quartiles | 1.02 (0.60-1.73)  1.14 (0.83-1.57) | 0.34  0.40 |
| Khalili, 2020^26^ | Non refined/high fiber grains   - CD - UC   Fruits and Vegetables   - CD - UC   Legumes and nuts   - CD - UC | aHR | Quartiles | 0.78 (0.55-1.10)  0.90 (0.70-1.40)  0.83 (0.60-1.15)  0.91 (0.74-1.13)  0.70 (0.49-0.98)  1.15 (0.94-1.41) | NS  NS  NS  NS  NS  NS |
| Lopes, 2022^58^ | Fiber  CD  UC  Fruits +vegetables  CD  UC | aHR | Quintiles | 0.67 (0.41-1.11)  0.97 (0.62-1.52)  0.82 (0.58-1.15) | 0.61  0.78  0.15 |
| Lopes, 2025^34^ | Nut Intake  Legume Intake | aHR  -CD  -UC  -CD  -UC | Quartiles | 0.96 (0.63, 1.47)  1.30 (0.92, 1.84)  1.26 (0.78, 2.04)  0.72 (0.44, 1.18) | 0.57  0.36  0.59  0.20 |
| Meyer, 2024^18^ | Total carbohydrates   - CD - UC   Sugars   - CD - UC   Fiber  - CD  - UC  Fruits, vegetables, legumes, nuts  - CD  - UC | aHR | Quartiles | 0.90 (0.45−1.81)  1.46 (0.94−2.27)  0.70 (0.40-1.21)  1.27 (0.91-1.79)  **0.49 (0.28-0.85)**  1.13 (0.81-1.58)  **0.50 (0.30-0.83)**  0.97 (0.71-1.33) |  |
| Narula, 2021 (BMJ)^29^ | Starch   - IBD - CD - UC   **Soft drink**   - **IBD** - **CD** - **UC**   **Sweets**   - **IBD** - **CD** - **UC**   Fruits, vegetables and legumes   - IBD | aHR | Tertiles | 1.50 (0.95-2.38)  **1.94 (1.42-2.66)**  **2.36 (1.23-4.55)**  **1.84 (1.29-2.64)**  **2.58 (1.44-4.62)**  **1.17 (0.35-3.84)**  **3.08 (1.57-6.05)**  1.31 (0.77-2.22) | 0.16  **0.001**  **0.003**  0.11 |
| Rubin, 2020^17^ | Fiber  IBD | aHR | Tertiles | 1.2 (0.91-1.60) *lowest vs highest* | NS |

| **Supplementary table 5. Results of included studies for fat exposure** | | | | | |  |
| --- | --- | --- | --- | --- | --- | --- |
| **Fat** | | | | | | |
| **Author (year)^reference^** | **Exposure** | **Measure** | **Dietary analyses** | **Risk measure** | **P trend** | |
| Ananthakrishnan, 2014^41^ | Total fat   - CD - UC   SFA   - CD - UC   Trans unsaturated fats   - CD - UC   MUFA   - CD - UC   PUFA   - CD - UC   n-3 PUFA   - CD - UC   n-6 PUFA   - CD - UC   **N-3/n-6 PUFA ratio**   - CD - **UC**   Arachidonic acid   - CD - UC   Linoleic acid   - CD - UC   Oleic acid   - CD - UC   Long chain n-3 PUFA   - CD - UC | aHR | Quintiles | 0.98 (0.66-1.45)  0.96 (0.68-1.35)  0.79 (0.53-1.19)  0.84 (0.59-1.19)  0.75 (0.50-1.13)  1.34 (0.94-1.92)  1.02 (0.69-1.49)  1.04 (0.74-1.46)  0.95 (0.63-1.42)  1.02 (0.72-1.47)  Not shown  0.88 (0.63-1.24)  Not shown  1.08 (0.77-1.52)  0.85 (0.55-1.30)  **0.69 (0.49-0.98)**  0.80 (0.55-1.18)  0.90 (0.65-1.27)  1.05 (0.7-1.56)  1.04 (0.73-1.48)  1.06 (0.72-1.55)  1.00 (0.70-1.41)  0.83 (0.57-1.23)  0.72 (0.51-1.01) | 0.71  0.94  0.16  0.82  0.18  0.07  0.82  0.61  0.41  0.69  Not shown  0.45  Not shown  0.59  0.33  **0.03**  0.18  0.63  0.97  0.71  0.84  0.55  0.11  0.13 | |
| Chan, 2014 (APT)^40^ | *CD only*  Total fat  Combined long chain fatty acids (DHA and EPA)  **Docosohexaenoic acid**  Eicosopentaenoic acid  Alpha linolenic Acid  Linoleic acid  Oleic acid | aOR | Quintiles | 1.48 (0.28–7.73)  1.03 (0.28–3.85)  **0.07 (0.02–0.81)**  6.43 (0.72–57.90)  0.38 (0.07–1.98)  2.34 (0.65–8.42)  1.94 (0.35–10.64) | 0.32  0.84  **0.04**  0.11  0.35  0.61  0.37 | |
| De Silva, 2014^39^ | Arachidonic acid  Oleic acid | aOR | Tertiles | 3^rd^ vs 1^st^ tertile  6.09 (1.05–35.23)  0.03 (0.00–0.56) | 0.10  0.03 | |
| Fu, 2024^28^ | Fish oil supplement  -CD  -UC  -IBD | aHR | Halves  (Yes/No)  Quintiles | **0.78 (0.64-0.94)**  **0.86 (0.75-0.98)**  **0.71 (0.47-1.08)** | **0.01**  **0.02**  **0.04** | |
| Hart, 2008^33^ | Polyunsaturated fatty acids, % of  total energy intake | OR | Quartiles | UC- 1.19 (0.99–1.43) | 0.07 | |
| Hart, 2009^38^ | *UC only*  **Docosohexaenoic acid**  Eicosopentaenoic acid  Alpha linolenic acid  Linoleic acid  Oleic acid |  |  | **0.23 (0.06–0.97)**  2.58 (0.66–10.05)  1.28 (0.46–3.58)  2.31 (0.99–5.36)  0.78 (0.26–2.40) | **0.03**  0.16  0.40  0.08  0.67 | |
| Huang, 2022^43^ | Fish oil  -IBD  -CD  -UC | aHR | Halves  (Yes/No) | **0.88 (0.78–0.99)**  0.88 (0.72–1.08)  **0.85 (0.75-0.99)** | **0.03**  0.22  **0.02** | |
| Jantchou, 2010^16^ | Total fat   - IBD - CD - UC | HR | Tertiles | 3^rd^ vs 1^st^ tertile  1.24 (0.57 – 2.72)  0.98 (0.25 – 3.88)  1.47 (0.56-3.84) | 0.77  0.88  0.34 | |
| John, 2010^42^ | Only UC  Total n-3 PUFAs  EPA  DHA | aOR | Tertiles | 3^rd^ vs 1^st^ tertile  0.31 (0.07–1.27)  0.25 (0.06–1.07)  **0.17 (0.04–0.78)** | 0.1  0.06  **0.02** | |
| Khalili, 2020^26^ | Olive or rapeseed oil   - CD - UC |  | Quartiles | 4^th^ vs 1^st^ quartile  0.70 (0.49-0.98) 0.89 (0.72-1.10) | -  NS | |
| Lopes, 2023^19^ | Only UC  n-3/n-6 ratio | aHR | Quintiles | 1.27 (0.92-1.75) | 0.10 | |
| Meyer, 2024^18^ | Total fat intake   - CD - UC   Saturated fatty acids   - CD - UC   Monounstaurated fatty acids   - CD - UC   Polyunsaturated fatty acids   - CD - UC | aHR | Quartiles | 1.13 (0.55−2.31)  0.89 (0.57−1.37)  1.78 (0.94-3.37)  0.73 (0.49-1.08)  0.68 (0.35−1.31)  0.98 (0.64−1.49)  0.72 (0.42−1.23)  1.11 (0.78−1.57) |  | |
| Narula, 2021 (BMJ)^29^ | **Fried food**   - **IBD** - **CD** - **UC** | aHR | Tertiles | 3^rd^ vs 1^st^ tertile  **3.02 (1.51-6.03)**  **1.19 (0.30-4.66)**  **3.76 (1.67-8.51)** | **0.006** | |
| Ye, 2024^23^ | Olive oil  CD  UC | aHR | Tertiles | 0.86 (0.67-1.11)  0.96 (0.83-1.06) |  | |

| **Supplementary table 6. Results of included studies for micronutrients exposure** | | | | | |
| --- | --- | --- | --- | --- | --- |
| **Author (year)^reference^** | **Exposure** | **Measure** | **Dietary analyses** | **Risk measure** | **P trend** |
| Ananthakrishnan, 2012^51^ | Predicted vit D   - CD - UC   Diet and suppl intake of vit D   - CD - UC | aHR | Quartiles | 0.55 (0.30–1.00)  0.68 (0.35–1.31)  0.76 (0.46–1.27)  0.64 (0.37–1.10) | **0.018**  0.17  0.22  **0.04** |
| Ananthakrishnan, 2015^44^ | Dietary Zinc   - **CD** - UC - Colonic CD - Ileal CD | aHR | Quartiles | **0.63 (0.43 – 0.93)**  1.40 (0.98 – 2.00)  **0.40 (0.19 – 0.85)**  1.23 (0.72 – 2.21) | **0.04** |
| Chen 2025 (CGH)^48^ | total antioxidant capacity | aHR  -CD  -UC | Quintiles | **0.66 (0.49, 0.90)**  0.85 (0.69, 1.06) | **0.009**  0.157 |
| Hart, 2008^33^ | \| Vitamin C, mg/day \| \| --- \| \| Vitamin D, microg/day \| \| Vitamin E, mg/day \| \| Carotene, microg/day \| \| Retinol, microg/day \| \| Calcium, mg/day \| \| Iron, mg/day \| | OR  UC only | Quartiles | \| 0.92 (0.76–1.10) \| \| --- \| \| 0.94 (0.75–1.20) \| \| 1.09 (0.87–1.36) \| \| 1.03 (0.84–1.25) \| \| 0.93 (0.75–1.14) \| \| 0.93 (0.75–1.15) \| \| 1.12 (0.88–1.43) \| | \| 0.35 \| \| --- \| \| 0.65 \| \| 0.45 \| \| 0.79 \| \| 0.47 \| \| 0.51 \| \| 0.37 \| |
| Jantchou, 2014^52^ | Calcium intake   - CD - UC   Dietary vit D   - CD - UC | aHR | Quartiles | 1.49 (0.60–3.68)  1.11 (0.49–2.52)  0.41 (0.14–1.24)  1.61 (0.61–4.23) | 0.39  0.73  0.13  0.38 |
| Khalili, 2016^46^ | Dietary Potassium   - **CD** - UC   Dietary Sodium   - CD - UC |  | Quintiles | **0.62 (0.40–0.95)**  0.74 (0.50–1.11)  1.32 (0.92–1.89)  1.04 (0.73–1.48) | **0.005**  0.08  0.44  0.77 |
| Khalili, 2017^15^ | Iron  CD  UC | aHR | Quintiles | 1.15 (0.76–1.74)  1.18 (0.79–1.76) | 0.67  0.35 |
| Kim, 2023 (Abstract)^60^ | sulfur microbial diet scores | HR | Quartiles | **CD : 1.40 (1.02- 1.92)**  UC : 1.17 (0.89- 1.55) | **0.04**  0.17 |
| Lu, 2017^47^ | Polyphenols   - CD - UC   **Flavones**   - **CD** - UC   Resveratrol   - **CD** - UC | OR  (case control) | Quartiles | 0.70 (0.33–1.49)  1.52 (0.88–2.63)  **0.61 (0.28–1.30)**  1.37 (0.81–2.34)  **0.40 (0.20–0.82)**  1.37 (0.81–2.34) | 0.17  0.16  **0.03**  0.14  **0.02**  0.14 |
| Lu, 2024^49^ | Quercetin  (daily intake from diet)  -IBD  -CD  -UC | aHR | Quintiles | **0.76 (0.60, 0.95)**  0.95 (0.62, 1.45)  **0.69 (0.53, 0.91)** | **0.004**  0.765  **0.001** |
| Nguyen, 2020^59^ | sulfur microbial diet scores | RR | Quartiles | CD : 1.49 (1.07- 2.08)  UC : 0.94 (0.7-1.26) | **0.01**  0.41 |
| Opstelten, 2016^22^ | Calcium intake   - CD - UC | aOR | Quartiles | 0.63 (0.28-1.42)  0.81 (0.49-1.34) | 0.23  0.60 |
| Opstelten, 2018^53^ | Serum Vit D   - CD - UC   Dietary of vit D   - CD - UC | OR (case control) | Quartiles | 0.69 (0.29–1.60)  1.22 (0.67–2.20)  1.08 (0.41–2.85)  1.15 (0.58–2.29) | 0.34  0.66  0.39  0.83 |
| Sauk, 2017^61^ (Abstract) | tryptophan intake | HR | Quintiles | CD : 0.70 (0.47-1.04)  UC : 0.91(0.64-1.29) | 0.09  0.82 |
| Sun, 2024^50^ | anthocyanin intake | HR | Quartiles | CD : 0.97 (0.66, 1.41)  **UC : 0.65 (0.51, 0.84)** | 0.536  **< 0.001** |
| Vasseur, 2020^45^ | Dietary Zinc   - **CD** - UC | RR | Tertiles | **0.12 (0.02–0.73)**  0.72 (0.26–2.00) | **0.02**  0.50 |
| Narula, 2021 (BMJ)^29^ | Urinary sodium  IBD  **Salty food/snack**  **IBD**  **CD**  **UC** | aHR | Tertiles | 0.99 (0.62-1.59)  **2.06 (1.41-3.00)**  **1.73 (0.80-3.73)**  **2.16 (1.40-3.33)** | 0.45  **0.009** |

| **Supplementary table 7. Results of included studies for alcohol exposure** | | | | | |
| --- | --- | --- | --- | --- | --- |
| **Author (year)^reference^** | **Exposure** | **Measure** | **Dietary analyses** | **Risk measure** | **P trend** |
| Bergmann, 2017^54^ | Alcohol use at recruitment   - CD - UC   Lifetime average alcohol use  - CD  - UC | aHR | Quintiles | 0.43 (0.13–1.47) 0.95 (0.52–1.76)  0.57 (0.06–5.20) 1.70 (0.80–3.58) |  |
| Casey, 2022^55^ | Alcohol   - CD - UC   **Beer**   - **CD** - UC   Liquor   - CD - UC | aHR | Quartiles | 0.84 (0.56, 1.24)  1.08 (0.77, 1.51)    **0.66 (0.34, 1.25)**  0.96 (0.61, 1.50)  1.28 (0.82, 2.00)  **1.48 (1.01, 2.16)** | 0.455  0.745  **0.05**  0.497  0.565  **0.045** |
| Liu, 2022^56^ | Red wine IBD  White wine  Beer+cider IBD  Spirits | OR | Quartiles | **0.86 (0.78–0.95)**  1.04 (0.93-1.15)  0.92 (0.82–1.03)  1.03 (0.93-1.14) | **0.004**  0.51  0.16  0.60 |

| **Supplementary table 8. Results of included studies for other foods/nutrients exposure** | | | | | | |
| --- | --- | --- | --- | --- | --- | --- |
| **Author (year)^reference^** | **Exposure** | **Measure** | **Dietary analyses** | | **Risk measure** | **P trend** |
| Jiang, 2024^57^ | Total AGE   - CD - UC   CML   - CD - UC   CEL   - CD - UC   MH-H1   - CD - UC | aHR | Tertiles | | 1.32 (0.94-1.86)  1.01 (0.80-1.26)  1.35 (0.95-1.92)  1.01 (0.81-1.27)  1.36 (0.96-1.93)  1.01 (0.81-1.27)  1.29 (0.92-1.81)  1.07 (0.85-1.35) | 0.065  0.979  0.098  0.942  0.064  0.933  0.079  0.584 |
| Lopes, 2022^58^ | Gluten   - CD - UC | aHR | Quintiles | 1.16 (0.82–1.64) 1.04 (0.75–1.44) | | 0.41  0.64 |
| AGE, advanced glycation end product; CEL, Nε-­ (1-­ carboxyethyl) lysine; CML, Nε­(carboxymethyl) lysine; MG-­ H1, Nd-­ (5-­ hydro-­ 5-­ methyl-­ 4-­ imidazolon-­ 2-­ yl)-­ ornithine; | | | | | | |

| **Supplementary table 9. Results of included studies for Mediterranean score exposure** | | | | | |
| --- | --- | --- | --- | --- | --- |
| **Author (year)^reference^** | **Exposure** | **Measure** | **Dietary analyses** | **Risk measure** | **P trend** |
| Khalili, 2020^26^ | **Med Score**   - **CD** - UC | aHR | Quartiles | 4^th^ vs 1^st^ tertile  **0.42 (0.22 – 0.78**)  1.08 (0.74 – 1.58) | **0.03**  0.61 |
| Khalili, 2023^63^ | **Med Score**  **CD**  UC | aHR | Quartiles | **0.58 [0.32, 1.06]**  1.00 [0.70, 1.42] | **0.04**  0.428 |
| Peters, 2022^10^ | Med Score   - CD - UC | OR |  | 0.98 (0.86-1.13)  1.06 (0.97-1.16) | 0.831  0.219 |
| Racine, 2016^62^ | Med Score   - CD - UC | IRR | Quintiles | 0.90 (0.45-1.77)  0.79 (0.48-1.32) | 0.67  0.41 |
| Ye, 2024^23^ | **MIND diet score***  (Per 3-point increment)   - IBD - CD - UC | aHR | Tertiles | **0.74 (0.62, 0.90)**  **0.66 (0.47, 0.94)**  **0.78 (0.62, 0.98)** | **0.002**  **0.022**  **0.031** |
| Xia, 2024^64^ | Alternate Mediterranean Diet (AMED) | aHR  -CD  -UC | Tertiles | **0.49 (0.31, 0.77)**  0.82 (0.64, 1.07) | **0.024**  0.155 |

*MIND : Mediterranean Dietary Approaches to Stop Hypertension Intervention for Neurodegenerative Delay

| **Supplementary table 9bis. Definitions and scorings of Mediterranean diets in the three articles included in the meta-analysis** |
| --- |

| Articles | Favorable | Unfavorable | Maximum scoring |
| --- | --- | --- | --- |
| Racine, 2016^62^ | Vegetables  Legumes  Fruits and nuts  Cereal products  Shellfish and seafood Monounsaturated to saturated fatty acids ratio  Moderate alcohol consumption:  10-50 g/d in men  5-25 g/d in women | Meat and meat products  dairy products  Low or high alcohol consumption | 9 |
| Khalili, 2023^63^ | Fruits and vegetables  Legumes  Non-refined/high-fiber grains Fermented dairy products,  Fish,  Olive or rapeseed oil  Alcohol : moderate consumption | Red or processed meat  Alcohol : <5 g/d or > 15 g/d | 8 |
| Xia, 2024^64^ | Vegetables  Legumes  Fruits  Nuts  Whole grains  Fish  MUFA:SFA ratio | Red and processed meat  Alcohol : <5 g/d or > 15 g/d | 9 |

| **Supplementary table 10. Results of included studies for inflammatory diet exposure** | | | | | | |
| --- | --- | --- | --- | --- | --- | --- |
| **Author (year)^reference^** | **Exposure** | **Measure** | **Dietary analyses** | **Risk measure** | **P trend** |  |
| Guevara, 2021^68^ | ISD score   - **CD** - UC | IRR | Quartiles | 1.71 (1.05–2.80)  0.89 (0.66–1.19) | **0.031**  0.436 |  |
| Lo, 2020^67^ | **High EDIP Score**   - **CD** - UC   Low to high   - CD - UC   High to low^a^   - CD - UC   High to high^a^   - CD - UC   Ileocolonic CD  **Ileal CD**  Colonic CD | aHR | Quartiles | 4^th^ vs 1^st^ tertile  **1.51(1.10-2.08)**  1.03 (0.77 – 1.36)  2.02 (1.09-3.75)  1.02 (0.56-1.86)  1.50 (0.76-2.98)  0.72 (0.37-1.39)  1.77 (1.10–2.84)  0.91 (0.60-1.38)  1.28 (0.71–2.29)  1.85 (1.01–3.37)  1.27 (0.80–2.00) | **0.01**  0.64  0.18  **0.03**  0.54 |  |
| Meyer, 2025^14^ | ISD score   - CD - UC | aHR | Quartiles | 1.88 (1.14-3.10)  0.85 (0.63-1.15) | <0.01  0.21 |  |
| Narula, 2021 (Gastroenterology)^66^ | EDIP score   - CD - UC | OR | Quartiles | 1.86 (0.78–4.39)  0.71 (0.41–1.24) | 0.203  0.495 |  |
| EDIP: Empirical dietary in ammatory pattern, ISD: Inflammatory Score of the Diet | | | | | |  |

| **Supplementary table 11. Results of included studies for healthy dietary index exposure** | | | | | |
| --- | --- | --- | --- | --- | --- |
| **Author (year)^reference^** | **Exposure** | **Measure** | **Dietary analyses** | **Risk measure** | **P trend** |
| Chen, 2025 (Lancet Reg Heal - Eur)^73^ | **Plant-based diet indexes** | aHR  IBD:UK biobank  IBD:EPIC | Quintiles | **0.75 (0.60, 0.94)**  **0.71 (0.59, 0.85)** | **0.003**  **0.0002** |
| Daher, 2025^72^  (abstract) | Vegan diet | OR | Yes/no | 1.14 (0.68, 1.78) |  |
| Fu, 2022 (Nutrients)^25^ | **Cardioprotective**  **Diet**   - **CD** - **UC** | aHR | Quintiles | **0.72 (0.55–0.95)**  **0.79 (0.65–0.95)** | **0.002**  **0.014** |
| Khalili, 2023^63^ | AHEI   - CD - UC   HEI 2015   - CD - UC   **HPDI**   - **CD** - UC | aHR | Quartiles | 0.73 [0.48, 1.12]  0.94 [0.71, 1.24]  0.90 [0.57, 1.41]  0.82 [0.60, 1.11]  **0.52 [0.32, 0.85]**  0.84 [0.63, 1.14] | 0.123  0.80  0.736  0.362  **0.01**  0.205 |
| Meyer, 2024^18^ | **FSAm NPS**  **CD**  UC | aHR | Quartiles | **2.04 (1.24–3.36)** 0.91 (0.69–1.21) | **<0.01**  0.76 |
| Peters, 2022^10^ | Protein score   - CD - UC   **LLDS**   - **CD** - UC   HEI   - CD - UC | OR |  | 0.93 (0.86-1.00)  1.02 (0.97 -1.07)  **0.95 (0.92-0.99)**  0.99 (0.96-1.01)  0.99 (0.97-1.01)  1.01 (0.99-1.02) | 0.062  0.483  **0.009**  0.310  0.371  0.421 |
| Sun, 2022^70^ | Unhealthy diet  CD  UC | aHR | Yes/No | **1.22 (1.04–1.43)**  **1.16 (1.04–1.29)** | **0.016**  **0.006** |
| Xia, 2024^64^ | Healthy Eating Index 2015 (HEI-2015)  Healthful Plant-based Diet Index (HPDI)  EAT-Lancet | -CD  -UC  -CD  -UC  -CD  -UC | Tertiles | **0.65 (0.47, 0.90)**  0.86 (0.69, 1.07)  1.24 (0.90, 1.72)  1.08 (0.87, 1.34)  1.09 (0.80, 1.48)  1.02 (0.83, 1.27) | **0.032**  0.124  0.958  0.714  0.909  0.721 |
| Yang, 2024^69^ | Healthy diet score  IBD  CD  UC | aHR | Per each SD increase | **0.94 (0.89, 0.99) 0.92 (0.84, 1.00)**  0.95 (0.90, 1.01) |  |
| Ye, 2025^71^ | EAT-Lancet diet score | aHR  -CD  -UC | Quartiles | **0.79 (0.62, 1.02)**  0.88 (0.73, 1.06) | **0.034**  0.085 |
| - FSAm NPS : Food Standards Agency modified nutrient profiling system () - Protein Score : higher intake of plant-derived protein relative to animal derived protein, is associated with improved health outcomes, including a lower likelihood of developing IBD. - The LifeLines Diet Score [LLDS] is a population-specific diet quality score that has been based on top-10 most prevalent diseases. - Healthy Eating Index [HEI] - Healthy diet is inspired from these 2 papers : Mozaffarian D, Appel LJ, Van Horn L. Components of a cardioprotective diet: new insights. Circulation 2011;123:2870-91. And US Department of Health and Human Services. 2015-2020 Dietary guidelines for Americans. 8th edition. December 2015. - Healthy diet score : Beydoun HA, Beydoun MA, Meirelles O, Erickson LD, Gamaldo AA, Weiss J, et al. Cardiovascular health, infection burden, and incident dementia in the UK   Biobank. Alzheimers Dement. 2023;19(10):4475–87 | | | | | |

| **Supplementary table 12. Results of included studies for food processing exposure** | | | | | |
| --- | --- | --- | --- | --- | --- |
| **Author (year)^reference^** | **Exposure** | **Measure** | **Dietary analyses** | **Risk measure** | **P trend** |
| Chen, 2023^74^ | Ultra-processed foods  CD  UC |  | Quintiles | 2.00 (1.32-3.03)  0.91 (0.70-1.18) | 0.001  0.473 |
| Lo, 2022^75^ | Ultra-processed foods  CD  UC  Unprocessed/minimally processed foods  CD  UC | aHR | Quartiles | 4^th^ vs 1^st^ tertile  1.70 (1.23-2.35)  1.20 (0.91-1.58)  0.78 (0.57-1.06)  0.80 (0.61-1.04) | 0.0008  0.25  0.14  0.08 |
| Meyer, 2023^76^ | Ultra-processed foods  CD  UC  Unprocessed/minimally processed foods  CD  UC |  | Quartiles | 1.48 (0.79-2.77)  0.93 (0.61-1.42)  0.57 (0.35-0.93)  0.89 (0.65-1.21) | 0.20  0.75  <0.01  0.43 |
| Narula, 2021 (BMJ)^29^ | Ultra-processed foods  CD  UC |  |  | 1.31 (0.63-2.73)  1.89 (1.32-2.71) |  |
| Vasseur, 2021^77^ | Ultra-processed foods   - IBD | RR | Tertiles | 3^rd^ vs 1^st^ tertile  1.44 (0.70–2.94) | 0.30 |

| **Supplementary table 13. Results of included studies for a posteriori dietary pattern exposure** | | | | | | |
| --- | --- | --- | --- | --- | --- | --- |
| **Author (year)^reference^** | **Exposure** | **Measure** | **Dietary analyses** | **Risk measure** | **P trend** |  |
| Khalili, 2014  (Abstract)^78^ | Western CD  Prudent CD  UC NA | aHR | Quintiles | 1.07 (0.63-1.80)  0.99 (0.56-1.74) | 0.56  0.26 |  |
| Peters, 2022^10^ | Dietary pattern 1   - CD - UC   Dietary pattern 2   - **CD** - UC   Dietary pattern 3   - CD - UC   Dietary pattern 4   - CD - UC   Dietary pattern 5   - CD - UC | OR |  | 1.00 (0.90-1.11)  1.00 (0.93-1.06)  1.16 (1.03-1.30)  1.01 (0.92-1.10)  0.99 (0.86-1.13)  1.11 (1.01-1.20)  1.01 (0.88-1.14)  1.02 (0.94-1.11)  0.90 (0.77-1.04)  1.01 (0.92-1.12) | 0.981  0.941  **0.013**  0.847  0.853  0.023  0.921  0.570  0.144  0.805 |  |
| Racine, 2016^62^ | **Sugar and soft drinks pattern**   - **UC**   Sugar, soft drinks, vegetables, and legumes pattern   - UC   Potatoes and seafood pattern   - UC   Vegetable pattern   - CD   Sugar and soft drinks pattern   - CD   Animal fats, seafood, potatoes, and alcohol pattern   - CD | aHR | Quintiles | ^5th^ vs 1^st^ tertile  **1.68 (1.00–2.84)**  1.17 (0.65–2.09)  0.77 (0.41–1.44)  1.03 (0.48–2.20)  1.48 (0.60–3.61)  0.71 (0.29–1.73) | **0.02**  0.66  0.62  0.91  0.93  0.32 |  |
| Song, 2024^21^ | **Traditional Northern pattern**   - **UC**   **Modern pattern**   - **UC**   **Spicy food**   - **UC** | aHR | Quartiles  Quintiles | **2.79 (1.93–4.05)**  **2.48 (1.63–3.78)**  **0.63 (0.45–0.88)** | **<0.001**  **<0.001**  **0.001** |  |
| Vasseur, 2021^77^ | IBD (UC and CD NA)  Western  Traditional  Healthy | RR | Tertiles | 1.24 (0.58-2.65)  0.99 (0.45-2.17)  0.78 (0.39-1.56) | 0.52  0.97  0.49 |  |
| Northern pattern high in wheat, low in rice  Modern pattern : high in meat and fruits  Pattern 1 : high in cooking oils, fats, grain products, potatoes, sugar, cakes, confectionery, condiments and sauces, dairy, and processed meat.  Pattern 2 : high in snacks, prepared meals, non-alcoholic beverages, condiments and sauces, low in vegetables fruit consumption.  Pattern 3 : high in red meat, poultry, processed meat  Pattern 4 : high in coffee, alcoholic beverages, low in tea.  Pattern 5: high in fish, eggs, nuts, vegetables, legumes, alcoholic beverages, soups, and fruits. | | | | | |  |

| **Supplementary table 14. Results of included studies for early life diet exposure** | | | | | |
| --- | --- | --- | --- | --- | --- |
| **Author (year)^reference^** | **Exposure** | **Measure** | **Dietary analyses** | **Risk measure** | **P trend** |
| Agrawal, 2024^81^ | breastfeeding duration | HR | breastfed >=6 months compared with 4–5 months | CD: 1.14 (0.77- 1.69)  UC: 1.16 (0.72- 1.86) | NA |
| De Freitas, 2025^85^ (abstract) | Diet during pregnancy  Lean fish  N-3-PUFA | HR  IBD  IBD |  | **0.65 (0.43, 0.98)**  **0.62 (0.38-0.98)** |  |
| Anneberg, 2025^84^ (abstract) | Diet during pregnancy  Diverse dietary pattern vs Western-like dietary pattern | HR  CD  UC |  | **0.41 (0.22, 0.76)**  0.73 (0.38, 1.39) |  |
| Guo, 2024 (IBD)^80^ | Diet diversity at 1 yr   - IBD - CD - UC   Diet diversity at 3 year   - IBD - CD - UC | aHR | Tertiles | ^5th^ vs 1^st^ tertile  0.96 (0.81-1.14)  1.10 (0.70-1.72)  0.90 (0.57-1.50)  0.98 (0.67-1.42)  1.17 (0.65–2.09)  0.94 (0.43-2.06) |  |
| Guo, 2024 (Gut)^83^ | **HEI**   - **IBD** - CD - UC   Meat   - IBD - CD - UC   **Fish**   - **IBD** - CD - **UC** - **UC 3 years**   Dairy   - IBD - CD - UC   Fruits   - IBD - CD - UC   Vegetables   - IBD - CD - UC   Grains   - IBD - CD - UC   Potatoes   - IBD - CD - UC   Sugar and fat-dense foods   - IBD - CD - UC   **Sugar-sweetened beverages**   - **IBD** - CD - UC | aHR | Tertiles | 3rd vs 1 st tertile  **0.75 (0.56-1.00)**  0.70 (0.33-1.45)  0.89 (0.36-2.24)  0.94 (0.70-1.27)  0.89 (0.84-1.50)  0.94 (0.39-2.31)  **0.70 (0.49-1.00)**  0.67 (0.39-1.17)  **0.46 (0.21-0.99)**  **0.46 (0.24-0.90)**  1.16 (0.89-1.53)  1.62 (0.85-3.11)  0.85 (0.52-1.40)  0.96 (0.48-1.92)  0.83 (0.40-1.69)  1.24 (0.55-2.81)  0.77 (0.58-1.03)  0.70 (0.27-1.81)  0.96 (0.24-3.92)  0.94 (0.67-1.32)  0.84 (0.55-1.26)  1.41 (0.38-5.22)  0.84 (0.59-1.20)  0.74 (0.17-3.15)  0.54 (0.29-1.03)  1.05 (0.78-1.41)  1.09 (0.44-2.71)  0.92 (0.55-1.53)  **1.42 (1.05-1.90)**  2.10 (0.56-7.88)  0.92 (0.25-3.26) |  |
| Guo, 2024 (Am J Clin Nutr)^79^ | Maternal diet diversity in pregnancy | HR | Tertiles | IBD: 0.75 (0.55-1.02)  CD: 0.81 (0.51- 1.28)  **UC: 0.46 (0.25-0.87)** | NA |
| Thompson, 2000^82^ | Breastfeeding | OR | Yes/no | CD: 0.4 (0.15-1.03)  UC: 2.76 (0.86-9.81) | NA |

**Supplementary references**

74 Chen J, Wellens J, Kalla R, *et al.* Intake of Ultra-processed Foods Is Associated with an Increased Risk of Crohn’s Disease: A Cross-sectional and Prospective Analysis of 187 154 Participants in the UK Biobank. *J Crohns Colitis* 2023; **17**: 535–52.

75 Lo CH, Khandpur N, Rossato SL, *et al.* Ultra-processed Foods and Risk of Crohn’s Disease and Ulcerative Colitis: A Prospective Cohort Study. *Clin Gastroenterol Hepatol* 2022; **20**: e1323–37.

76 Meyer A, Dong C, Casagrande C, *et al.* Food Processing and Risk of Crohn’s Disease and Ulcerative Colitis: A European Prospective Cohort Study. *Clin Gastroenterol Hepatol* 2023; **21**: 1607-1616.e6.

77 Vasseur P, Dugelay E, Benamouzig R, *et al.* Dietary Patterns, Ultra-processed Food, and the Risk of Inflammatory Bowel Diseases in the NutriNet-Santé Cohort. *Inflamm Bowel Dis* 2021; **27**: 65–73.

78 Hamed K, Ashwin A, Jenny S, Leslie H, James R, Andrew C. P-028 Dietary Patterns and Risk of Crohn’s Disease and Ulcerative Colitis. *Inflamm Bowel Dis* 2014; **20**: S36–7.

79 Guo A, Brantsæter AL, Borge TC, *et al.* Maternal diet in pregnancy and the risk of inflammatory bowel disease in the offspring: a prospective cohort study. *Am J Clin Nutr* 2024. DOI:10.1016/J.AJCNUT.2024.10.017.

80 Guo A, Ludvigsson J, Hård af Segerstad EM, *et al.* Early-Life Diet Diversity and the Subsequent Risk of Inflammatory Bowel Disease: Findings From Two Scandinavian Birth Cohorts. *Inflamm Bowel Dis* 2024; published online Sept 13. DOI:10.1093/IBD/IZAE210.

81 Agrawal M, Størdal K, Vinkel Hansen A, *et al.* Breastfeeding Duration Is Not Associated With Offspring Inflammatory Bowel Disease Risk in Three Population-Based Birth Cohorts. *Clin Gastroenterol Hepatol* 2024; **22**. DOI:10.1016/J.CGH.2024.04.013.

82 Thompson NP, Montgomery SM, Wadsworth MEJ, Pounder RE, Wakefield AJ. Early determinants of inflammatory bowel disease: use of two national longitudinal birth cohorts. *Eur J Gastroenterol Hepatol* 2000; **12**: 25–30.

83 Guo A, Ludvigsson J, Brantsæter AL, *et al.* Early-life diet and risk of inflammatory bowel disease: a pooled study in two Scandinavian birth cohorts. *Gut* 2024; **73**: 590–600.

84 Anneberg MSc OMR, Halldórsson ÞI, Olsen SF, *et al.* P1223 Maternal dietary patterns during pregnancy and offspring’s risk of paediatric Inflammatory Bowel Disease - a cohort study. *J Crohn’s Colitis* 2025; **19**: i2213–i2213.

85 Brusco De Freitas M, Anneberg OM, Frodi Olsen S, *et al.* P1245 Maternal intake of fish, dietary n-3 polyunsaturated fatty acids, and fish oil supplements during pregnancy and risk of early childhood Inflammatory Bowel Disease in the offspring. *J Crohn’s Colitis* 2025; **19**: i2254–i2254.

86 Loughin TM. A systematic comparison of methods for combining p-values from independent tests. *Comput Stat Data Anal* 2004; **47**: 467–85.

87 Tapsell LC, Neale EP, Satija A, Hu FB. Foods, Nutrients, and Dietary Patterns: Interconnections and Implications for Dietary Guidelines. *Adv Nutr* 2016; **7**: 445–54.

88 Thacker N, Collins C.E, Duncanson K, et al. ANTIBIOTICS, URBAN ENVIRONMENT AND WESTERN DIET PATTERN INCREASE RISK OF PAEDIATRIC INFLAMMATORY BOWEL DISEASE: A META-ANALYSIS. Gastroenterology 2023; **164**: S-60.

89 Ge J, Han TJ, Liu J, *et al.* Meat intake and risk of inflammatory bowel disease: A meta-analysis. *Turk J Gastroenterol* 2015; **26**: 492–7.

90 Mallon K, McBride C, Doherty PG, Burns DR. P862 Dietary risk factors associated with onset of IBD: a systematic literature review and meta-analysis. *J Crohn’s Colitis* 2023; **17**: i985–6.

91 Milajerdi A, Ebrahimi-Daryani N, Dieleman LA, Larijani B, Esmaillzadeh A. Association of Dietary Fiber, Fruit, and Vegetable Consumption with Risk of Inflammatory Bowel Disease: A Systematic Review and Meta-Analysis. *Adv Nutr* 2021; **12**: 735–43.

92 Mozaffari H, Daneshzad E, Larijani B, Bellissimo N, Azadbakht L. Dietary intake of fish, n-3 polyunsaturated fatty acids, and risk of inflammatory bowel disease: a systematic review and meta-analysis of observational studies. *Eur J Nutr* 2020; **59**. DOI:10.1007/S00394-019-01901-0.

93 Piovani D, Danese S, Peyrin-Biroulet L, Nikolopoulos GK, Lytras T, Bonovas S. Environmental Risk Factors for Inflammatory Bowel Diseases: An Umbrella Review of Meta-analyses. *Gastroenterology* 2019; **157**: 647-659.e4.

94 Wang F, Lin X, Zhao Q, Li J. Fat intake and risk of ulcerative colitis: Systematic review and dose-response meta-analysis of epidemiological studies. *J Gastroenterol Hepatol* 2017; **32**: 19–27.

95 Zhou XL, Zhao QQ, Li XF, Li Z, Zhao SX, Li YM. Protein intake and risk of inflammatory bowel disease: A meta-analysis. *Asia Pac J Clin Nutr* 2022; **31**: 443–9.

96 Zeng L, Hu S, Chen P, Wei W, Tan Y. Macronutrient Intake and Risk of Crohn’s Disease: Systematic Review and Dose-Response Meta-Analysis of Epidemiological Studies. *Nutrients* 2017; **9**. DOI:10.3390/NU9050500.

97 Güngör D, Nadaud P, Dreibelbis C, *et al.* Never Versus Ever Feeding Human Milk and Inflammatory Bowel Disease: A Systematic Review [Internet]. 2019; published online April 15. DOI:10.52570/NESR.PB242018.SR0222.

98 Hou JK, Abraham B, El-Serag H. Dietary intake and risk of developing inflammatory bowel disease: a systematic review of the literature. *Am J Gastroenterol* 2011; **106**: 563–73.

99 Jin ZQ, Lu HG, Wu Q Bin, *et al.* A meta-analysis of dietary carbohydrate intake and inflammatory bowel disease risk: evidence from 15 epidemiology studies. *Rev Esp enfermedades Dig* 2019; **111**: 5–9.

100 Khademi Z, Pourreza S, Amjadifar A, Torkizadeh M, Amirkhizi F. Dietary Patterns and Risk of Inflammatory Bowel Disease: A Systematic Review of Observational Studies. *Inflamm Bowel Dis* 2024; **30**. DOI:10.1093/IBD/IZAD297.

101 Khademi Z, Milajerdi A, Larijani B, Esmaillzadeh A. Dietary Intake of Total Carbohydrates, Sugar and Sugar-Sweetened Beverages, and Risk of Inflammatory Bowel Disease: A Systematic Review and Meta-Analysis of Prospective Cohort Studies. *Front Nutr* 2021; **8**. DOI:10.3389/FNUT.2021.707795.

102 Khorshidi M, Djafarian K, Aghayei E, Shab-Bidar S. A posteriori dietary patterns and risk of inflammatory bowel disease: a meta-analysis of observational studies. *Int J Vitam Nutr Res* 2020; **90**: 376–84.

103 Li T, Qiu Y, Yang HS, *et al.* Systematic review and meta-analysis: Association of a pre-illness Western dietary pattern with the risk of developing inflammatory bowel disease. *J Dig Dis* 2020; **21**: 362–71.

104 Liu X, Wu Y, Li F, Zhang D. Dietary fiber intake reduces risk of inflammatory bowel disease: result from a meta-analysis. *Nutr Res* 2015; **35**: 753–8.

105 Narula N, Chang NH, Mohammad D, *et al.* Food Processing and Risk of Inflammatory Bowel Disease: A Systematic Review and Meta-Analysis. *Clin Gastroenterol Hepatol* 2023; **21**: 2483-2495.e1.

106 Jayasooriya N, Baillie S, Blackwell J, *et al.* Systematic review with meta-analysis: Time to diagnosis and the impact of delayed diagnosis on clinical outcomes in inflammatory bowel disease. *Aliment Pharmacol Ther* 2023; **57**: 635–52.

107 https://crohnsandcolitis.org.uk/media/n3udnuct/understanding-diagnostic-delays-in-crohns-and-colitis-final-report-22-03-22-1.pdf

108 Chan SSM, Chen Y, Casey K, *et al.* Obesity is Associated With Increased Risk of Crohn’s disease, but not Ulcerative Colitis: A Pooled Analysis of Five Prospective Cohort Studies. *Clin Gastroenterol Hepatol* 2022; **20**: 1048–58.

109 Neustaeter A, Lee S-H, Xue M, *et al.* A218 ASSOCIATIONS BETWEEN ADHERENCE TO LITERATURE-DERIVED DIETARY INDICES AND PRE-DISEASE BIOMARKERS: IMPLICATIONS FOR CROHN’S DISEASE PREVENTION. *J Can Assoc Gastroenterol* 2023; **6**: 55–55.

110 Julia C, Baudry J, Fialon M, *et al.* Respective contribution of ultra-processing and nutritional quality of foods to the overall diet quality: results from the NutriNet-Santé study. *Eur J Nutr* 2023; **62**: 157–64.

111 Mahid SS, Minor KS, Soto RE, Hornung CA, Galandiuk S. Smoking and inflammatory bowel disease: a meta-analysis. *Mayo Clin Proc* 2006; **81**: 1462–71.

112 Chatellier G, Zapletal E, Lemaitre D, Menard J, Degoulet P. The number needed to treat: a clinically useful nomogram in its proper context. *BMJ* 1996; **312**: 426–9.

113 Olivera PA, Martinez-Lozano H, Leibovitzh H, *et al.* Healthy First-Degree Relatives From Multiplex Families vs Simplex Families Have Higher Subclinical Intestinal Inflammation, a Distinct Fecal Microbial Signature, and Harbor a Higher Risk of Developing Crohn’s Disease. *Gastroenterology* 2025; **168**. DOI:10.1053/J.GASTRO.2024.08.031.

114 Rosato V, Temple NJ, La Vecchia C, Castellan G, Tavani A, Guercio V. Mediterranean diet and cardiovascular disease: a systematic review and meta-analysis of observational studies. *Eur J Nutr* 2019; **58**: 173–91.

115 Ruan X, Che T, Chen X, et al. Mendelian randomisation analysis for intestinal disease: achievement and future. *eGastroenterology*. 2024;2(2):e100058.

116 Takeuchi T, Nakanishi Y, Ohno H. Microbial Metabolites and Gut Immunology. *Annu Rev Immunol* 2024; **42**: 153–78.

117 Arpaia N, Campbell C, Fan X, *et al.* Metabolites produced by commensal bacteria promote peripheral regulatory T-cell generation. *Nature* 2013; **504**: 451–5.

118 Kim M, Qie Y, Park J, Kim CH. Gut Microbial Metabolites Fuel Host Antibody Responses. *Cell Host Microbe* 2016; **20**: 202–14.

119 Whelan K, Bancil AS, Lindsay JO, Chassaing B. Ultra-processed foods and food additives in gut health and disease. *Nat Rev Gastroenterol Hepatol* 2024; **21**: 406–27.

120 Chassaing B, Koren O, Goodrich JK, *et al.* Dietary emulsifiers impact the mouse gut microbiota promoting colitis and metabolic syndrome. *Nature* 2015; **519**: 92–6.

121 Viennois E, Bretin A, Dubé PE, *et al.* Dietary Emulsifiers Directly Impact Adherent-Invasive E. coli Gene Expression to Drive Chronic Intestinal Inflammation. *Cell Rep* 2020; **33**. DOI:10.1016/J.CELREP.2020.108229.
